# Uncovering dormancy stage predictors in sweet cherry through DNA methylation and machine learning integration

**DOI:** 10.3389/fpls.2025.1659345

**Published:** 2025-09-04

**Authors:** Gabriela M. Saavedra, Poliana Povea, Claudio Urra, José Gaete-Loyola, Carlos Maldonado, Andrea Miyasaka Almeida

**Affiliations:** ^1^ Centro de Genómica y Bioinformática, Facultad de Ciencias, Ingeniería y Tecnología, Universidad Mayor, Santiago, Chile; ^2^ Programa de Doctorado en Genómica Integrativa, Vicerrectoría de Investigación, Universidad Mayor, Santiago, Chile; ^3^ Escuela de Agronomía, Facultad de Ciencias, Ingeniería y Tecnología, Universidad Mayor, Santiago, Chile

**Keywords:** stage predictive model, epigenetics, biomarkers, *Prunus avium*, feature selection, bud break

## Abstract

**Background:**

*Prunus Avium* L. dormancy is a complex physiological process that allows floral outbreaks to survive adverse winter conditions and resume favorable spring growth. Traditional phenological evaluations and agroclimatic models, although widely used, exhibit limited resolution and robustness over the years and cultivars. Epigenetic mechanisms, particularly DNA methylation, have emerged as critical regulators of dormancy transitions. However, the integration of methylation data with automatic learning tools (ML) for predictive modeling remains largely unexplored in perennial species. This study presents an integrative frame that combines whole-genome bisulfite sequencing and supervised ML to identify methylation markers at the cytosine and region level associated with specific dormancy stages in the sweet cherry.

**Methods:**

DNA methylation data sets from three different experiments underwent classification using Random Forest (RF) and eXtreme Gradient Boosting (XGBoost), complemented by SHapley Additive exPlanations (SHAP) for interpretability. The importance of the features was evaluated using the Integrated Model consensus in the RF, XGBoost, and SHAP metrics.

**Results:**

The selection of features significantly improved the classification performance in the three-stages models (paradormancy, endodormancy, ecodormancy) and two-stages (endodormancy and ecodormancy). RF constantly exceeded XGBoost, achieving an accuracy of up to 97.1% in the two-stages scenario using informative cytosine level data. The SHAP analyses demonstrated that the selected feature effectively discriminated among stages of dormancy and revealed biologically significant epigenetic features. The key features were distributed not random throughout the genome, often colocalizing with transposable elements of long terminal repetition (LTR), particularly LTR/ty3-retrotransposons and LTR/copia families. Some features also co-localize with QTLs for chilling and heat requirement, flowering time and maturity date previously identified.

**Conclusions:**

This study highlights the usefulness of combining high-resolution methylation data with interpretable ML techniques to identify robust dormancy biomarkers. The enrichment of the features associated with dormancy within the transposable elements and the proximal regions of genes suggests an epigenetic regulation through the remodeling of chromatin mediated by TE. These findings contribute to a deeper understanding of dormancy mechanisms and offer a basis for the development of non-destructive tools based on methylation to improve phenological management in perennial fruit crops.

## 1 Introduction

Dormancy in fruit species such as cherry (*Prunus avium* L.) is an essential process that synchronizes flower bud growth and reproduction with seasonal cycles (Considine and Considine, 2016). The bud dormancy has been classified into three widely accepted phases: paradormancy, endodormancy, and ecodormancy, which respond to physiological and environmental signals, allowing buds to survive adverse winter conditions and resume development in spring (Cline and Deppong, 1999; Considine and Considine, 2016). The dormancy has generally been assessed using destructive phenological assays (e.g., forcing tests; Baumgarten et al., 2021) and agroclimatic models (chill hours, chill units, or chill portions; Luedeling et al., 2011; Baumgarten et al., 2021). However, these approaches have significant limitations in terms of accuracy and interannual application due to dynamically changing environmental conditions (Alonso-González et al., 2020). Therefore, several studies have sought molecular biomarkers that allow more accurate, rapid, and noninvasive monitoring of bud physiological status.

During the past decade, progress has been made in the study of the mechanisms associated with dormancy stages in various species, improving the general understanding of dormancy transitions in different species (Rothkegel et al., 2017; Yang et al., 2021; Rothkegel et al., 2020; Ding et al., 2024). These studies have revealed that dormancy phases are regulated by DORMANCY-ASSOCIATED MADS-box (DAM) genes, phytohormones, carbohydrates, temperature, photoperiod, reactive oxygen species, water deprivation, cold acclimation, and epigenetic regulation. Particularly, DNA methylation has emerged as one of the key epigenetic mechanisms involved in plant development and environmental responses. In this sense, dynamic changes in genome methylation patterns throughout the dormancy cycle, suggesting a functional role for this epigenetic mark in determining bud fate (Rothkegel et al., 2020; Yang et al., 2021; Kumar et al., 2016). In this sense, Rothkegel et al. (2017) indicated that in *Prunus avium* L, genome methylation patterns are one of the mechanisms that regulate the MADS-box genes controlling bud dormancy. Similarly, Kumar et al. (2016) showed that DNA methylation has been linked to chilling acquisition during dormancy in *Malus domestica*. Additionally, high levels of DNA methylation have been observed during the induction of endodormant floral buds in blueberry compared to those in the ecodormant stage (Li et al., 2015), which indicates a dynamic regulation throughout the dormancy stages.

The development of high-throughput sequencing technologies, such as whole-genome bisulfite sequencing (WGBS), enables the precise examination of methylation status at individual CpG sites with high resolution (Zhou et al., 2019). However, this approach generates highly complex and high-dimensional datasets, where the number of features (methylated sites) far exceeds the number of available samples. This problem, coupled with biological variability between cultivars and climatic heterogeneity between seasons, poses significant challenges for conventional statistical analyses and hampers biomarker discovery and straightforward biological interpretation (Zhang et al., 2018).

Artificial intelligence (AI)-based approaches have shown great promise for the analysis of complex and high-dimensional molecular data (Bzdok et al., 2018). However, one of the main limitations of these algorithms in biology is their interpretability and identification of biologically relevant variables. In this sense, feature selection is essential to eliminate noisy features, improve model performance, and optimize interpretability (Lundberg and Lee, 2017). Ensemble learning algorithms, such as Random Forest (RF) and eXtreme Gradient Boosting (XGBoost), have demonstrated exceptional performance in genomic studies due to their ability to handle noisy, imbalanced, and high-dimensional data while providing internal feature importance metrics (Raihan et al., 2023; Peng and Yu, 2024; Chu et al., 2024; Li et al., 2024; Zhang et al., 2024). Furthermore, the SHapley Additive exPlanations (SHAP) method offers a game-theoretic interpretation of each variable’s individual contribution to model predictions (Lundberg and Lee, 2017). These models (RF, XGB, and SHAP) have been successfully used to identify important variables in predictive models for human diseases (Raihan et al., 2023), agronomic traits (Zhang et al., 2024), precision agriculture (Li et al., 2024), and seed viability (Chu et al., 2024).

In this study, DNA methylation profiles obtained via WGBS with supervised Machine Learning (ML) algorithms (RF and XGB) and SHAP interpretability analyses were integrated to identify informative cytosines and methylated regions capable of discriminating between different dormancy stages in sweet cherry floral buds. Classification models were constructed for two scenarios: (i) three classes (paradormancy, endodormancy, and ecodormancy stages) and (ii) two classes (endodormancy and ecodormancy stages). This integrative strategy not only improves classification accuracy of dormancy stages but also provides deeper insights into the epigenetic mechanisms governing this process, offering potential biomarkers for breeding programs and more precise tools for phenological management in perennial fruit species.

## 2 Materials and methods

### 2.1 Plant material, sampling, and chilling requirement determination under forcing conditions

Adult (9–12-year-old) sweet cherry (*Prunus avium* L.) trees of cultivars Santina and Regina were sampled from two commercial orchards, “Morza” and “Entre Ríos”, located in the Maule and O’Higgins regions of Chile, respectively during autumn/winter of 2022 (Experiment 3, **Table 1**). As experiments were performed in growth chambers (Experiments 1 and 2, **Table 1**) the cold accumulation as chilling hours was calculated according to Weinberger (1950). To facilitate more comprehensible data comparison labels, all chilling accumulation measurements were transformed into chilling hours. A second experiment using Regina cultivar was also performed. Adult (8–10-year-old) sweet cherry ‘Regina’ trees, which were part of the INIA Sweet Cherry Breeding Program collection located at the Los Tilos Station, Metropolitan Region of Chile were used. Samples of corresponding branches bearing floral buds were obtained from this orchard at the beginning of autumn 2021 (April in the Southern hemisphere) and subjected to continuous chilling as described by Soto et al. (2022). Briefly, the collected branches were transported to the laboratory, disinfected, separated into lots of 4–5 branches, and stored at 4–6 °C for progressive chilling accumulation.

**Table 1:** Summary of evaluated cultivars, trial locations, required chilling hours per cultivar, chilling hours sampling, and year of the trial.

Cultivar	Fields	Chill requirement	Chill Hour (CH) sampling	Year	Experiment
Royal Dawn (RD)	Mostazal	350–500 CH	0 – 173 – 348 – 516	2015	Experiment 1
Kordia (K)	Quillota	1450–1600 CH	0 – 443 – 1295 - 1637	2015	Experiment 1
Santina (S)	Morza	600–800 CH	269 - 599 - 973 - 1234	2022	Experiment 3
Regina (R)	Los Tilos	1150-1600	200 – 1160 - 1700	2021	Experiment 2
	Entre Ríos (E)	770–830 CH	202 - 463 - 769 - 837 - 959	2022	Experiment 3
	Morza (M)	600–800 CH	268 - 599 - 973 - 1234	2022	Experiment 3

Floral buds were collected from each cultivar at different chill accumulation time points, frozen in liquid nitrogen, and transferred to our laboratory facilities. The dormancy status at each time point was assessed through forcing experiments. Bud break percentages were recorded during 14 days at 25°C under a 16/8 h day/night photoperiod. After 14 days, the phenological status of floral buds was scored, and the chilling requirement was considered fulfilled when at least 50% of the buds burst (BBCH 51 stage; Fadón et al., 2015).

### 2.2 Yield and quality analysis of isolated DNA and sequencing

DNA was extracted from 100 mg of ground frozen tissue using the DNeasy Plant Mini Kit (Qiagen) following the manufacturer’s procedure. The DNA eluted in 50 µl of water was quantified by Qubit DNA High Sensitivity fluorometry assay (Life Technologies). Its integrity was evaluated on a 0.8% agarose gel. For library preparation, a 150 bp paired-end sequencing strategy was conducted by Novogene (USA). Library preparation and sequencing were performed in an Illumina Novaseq System to generate ~ 20 Gbp of data per sample.

### 2.3 BS-seq datasets and processing

**Figure 1:**
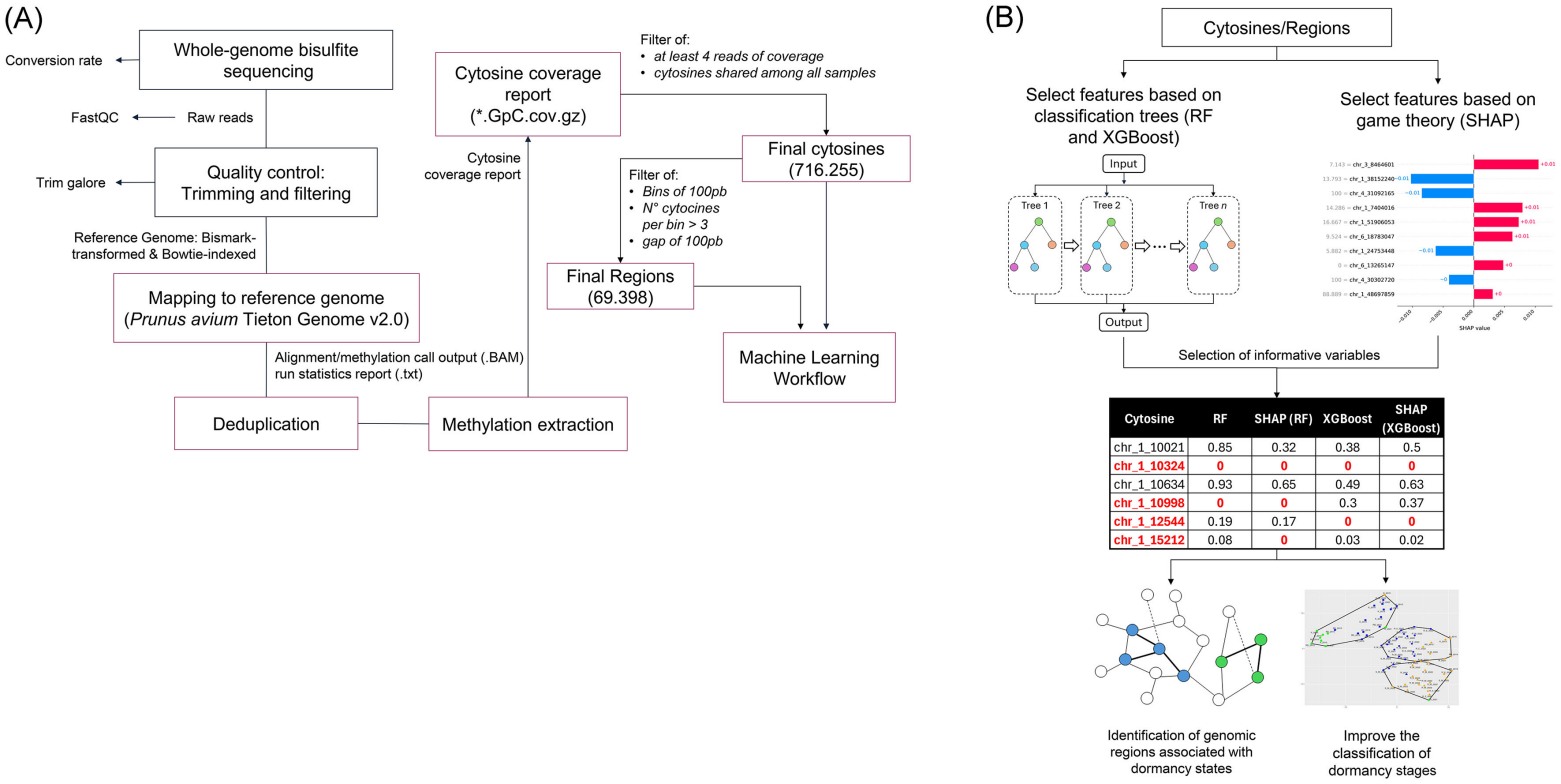
Workflows. Bisulfite sequencing analysis **(A)** and Machine Learning-based selection of informative features **(B)**.

Kordia and Royal Dawn cultivars’ raw data were retrieved from the SRA database (PRJNA610988 and PRJNA610989). These datasets collection and processing procedures were described by Rothkegel et al. (2020). Raw data quality analysis was performed on samples of Kordia (K_2015), Royal Dawn (RD_2015), Regina Los Tilos (R_2021), Regina Morza (R_M_2022), Regina Entre Ríos (R_E_2022) and Santina Morza (S_M_2022). Quality assessments were performed with FastQC v0.12.1 (Andrews, 2010) software and trimmed with Trim Galore (Krueger et al., 2016), which cuts adaptors and improves raw data quality (**Supplementary Table S1**). Clean bisulfite-treated data were then analyzed with the Bismark software (Krueger and Andrews, 2011), then mapped to *Prunus avium* Tieton v2.0 assembly as the reference genome (**Supplementary Table S2**). A deduplication step was required to remove duplicated alignments caused by excessive PCR amplification. Then, we proceed with the methylation extraction step to identify positions of every cytosine in the corresponding DNA methylation context: CG, CHG, and CHH (where H is A, C, or T) (**Supplementary Figure S1**). Datasets representing methylated cytosines and methylated genomic regions were generated following the BS-seq Analysis Workflow (**Figure 1A**).

CpG coverage was used to generate a cytosine database used as an ML algorithm input. In this database, filters were applied. For individual cytosines, consider at least 4 reads of coverage by cytosine and present among all samples, resulting in 716,255 cytosines. The filter applied by region was in the previously filtered cytosines, 4 or more cytosines were looked for within bins of 100 bp, with a gap of 100 bp between bins. This filter resulted in 69,398 regions. This filtered data was used as input for the classification analysis, using both RF and XGBoost as machine learning algorithms.

### 2.4 Classification and interpretation methodologies for variables

Two classification algorithms (RF and XGBoost) were employed to model the classification task. In addition, SHAP values were used to interpret the classification models, identifying the most informative features contributing to predictions (**Figure 1B**). The SHAP-derived feature importance scores were compared against the built-in feature importance metrics of both XGBoost and RF enabling a comprehensive evaluation of key features relevant to the classification task.

#### 2.4.1 Random forest

The RF algorithm was employed in this study due to its robustness, interpretability, and its capacity to process high-dimensional datasets that may contain irrelevant or noisy features. RF is an ensemble learning method that constructs a collection of decision trees using two key sources of randomness: (i) bootstrap sampling, which involves drawing random subsets of the training data with replacement, and (ii) random feature selection, where only a randomly chosen subset of features is considered at each decision node when splitting. This dual randomization strategy helps to reduce model variance and overfitting, improving generalization (Liu et al., 2012). For training, the original dataset is divided into in-bag samples (used to build individual trees) and out-of-bag samples, which serve as an internal validation set to assess model performance. Approximately two-thirds of the data are used for training, while the remaining third is reserved for out-of-bag error estimation, which provides an unbiased measure of predictive accuracy (Kavzoglu and Teke, 2022). Each decision tree in the ensemble outputs a class prediction, and the RF model aggregates these results to determine the final classification. This aggregation is done using a majority voting mechanism, formally defined as (Breiman, 1996):


$$H\left(x\right)=argarg\;\sum_{i=1}^{k}I\left(h_{i}\left(x\right)=Y\right)$$

where *H(x)* is the final prediction of the RF model for instance *x*, $h_{i}\left(x\right)$ is the prediction of the *i*-th decision tree, *I* is the indicator function, and *Y* represents a class label. This voting scheme ensures that the final decision reflects the consensus of the ensemble. In addition to its predictive capabilities, RF also provides an intrinsic measure of feature importance. The Gini index measures the decrease in node impurity contributed by each variable. A higher Gini importance score indicates a greater contribution to the classification process.

#### 2.4.2 XGBoost

The XGBoost algorithm was utilized in this study as a complementary classification method due to its high predictive performance and ability to handle noisy, high-dimensional biological data (Kavzoglu and Teke, 2022). Originally proposed by Chen and Guestrin (2016), XGBoost is based on the gradient boosting framework and builds models sequentially, where each subsequent tree attempts to correct the residual errors of the ensemble constructed so far. Unlike Random Forest, which builds trees independently, XGBoost optimizes performance by minimizing a regularized objective function in a stage-wise manner.

At each iteration t, the algorithm adds a new function ft to improve the model’s prediction. The overall objective function can be expressed as (Chen and Guestrin, 2016):

$L\left(\emptyset \right)=\sum_{i}l\left({\hat{y}}_{i},y_{i}\right)+\sum_{k}\Omega \left(f_{k}\right)$


$$\text{with Ω}\left(f\right)=\gamma T+\frac{1}{2}\lambda \|w{|}^{2}$$

where *l* is a differentiable convex loss function that measures the difference between the prediction ${\hat{y}}_{i}$ and the target $y_{i}$ . The regularization term *Ω* penalizes the complexity of the model, thereby controlling model complexity and reducing the risk of overfitting. For this, *T* is the number of leaf nodes in the tree, w is the score of each leaf, λ and γ are regularization parameters that control the penalty for model complexity. This formulation encourages the model to produce trees with fewer and more informative splits, improving generalization. XGBoost also implements strategies such as learning rate and column subsampling to further prevent overfitting.

#### 2.4.3 SHAP

To enhance model interpretability and ensure transparency in the classification process, this study applied SHAP as a *post hoc* interpretability method. SHAP, introduced by Lundberg and Lee (2017), is a unified framework rooted in cooperative game theory that quantifies the contribution of each feature to a model’s prediction. Unlike traditional feature importance scores generated by ensemble algorithms, SHAP values allow for both global and local interpretability, revealing not only which features are important but also how they positively or negatively influence specific predictions.

The SHAP methodology assigns an importance value φ*_i_* to each feature, representing the marginal contribution of including that feature in the prediction model, averaged over all possible feature subsets. Formally, the Shapley value is defined as:


$$\varphi_{i}=\sum_{S\subseteq F,\left\{i\right\}}\frac{\left|S\right|!\left(\left|F\right|-\left|S\right|-1\right)!}{\left|F\right|!}\left[f_{S\cup \;\left\{i\right\}}\left(x_{S\cup \;\left\{i\right\}}\right)-f_{S}\left(x_{S}\right)\right]$$

where *F* is the full set of input features, *S* is a subset of features not containing *i*, $f_{S\cup \left\{i\right\}}$ and $f_{S}$ are retrained, and predictions of these two models are compared to the current input $f_{S\cup \left\{i\right\}}$ ( $x_{S\cup \left\{i\right\}}$ )- $f_{S}\left(x_{S}\right)$ , where $x_{S}$ represents the values of the input features in the set *S*. This formulation ensures both local accuracy and consistency, two desirable properties for interpretable machine learning (Bi et al., 2020).

### 2.5 Feature selection via integrated model (RF, XGB, and SHAP)

To identify the most relevant and biologically informative features, a comprehensive feature selection strategy was implemented by integrating importance metrics from four distinct perspectives: RF, XGB, and SHAP computed over both RF and XGB models. Each method was used to independently estimate the relevance of input features based on either intrinsic model properties or *post hoc* explanations.

Feature importance scores were derived directly from the trained RF and XGB models using their respective built-in ranking mechanisms. In parallel, SHAP values were calculated to generate explanations for the predictions of both the RF and XGB models.

To ensure robustness and consistency, only features that exhibited non-zero importance across all four models: RF, XGB, SHAP(RF), and SHAP(XGB) were retained. This intersection-based filtering process served three main purposes: (i) to reduce the number of input features fed into the final models, (ii) to identify components with consistent predictive relevance and minimal redundancy, and (iii) to lower computational complexity and improve model generalizability. Additionally, by retaining only consistently informative methylation features, the models achieved improved interpretability while maintaining high classification performance in dormancy stage prediction.

All computations were carried out using Python (v3.12.7). RF and XGB models were implemented using the sklearn and xgboost libraries, while SHAP analyses were conducted via the SHAP library. All codes are provided in **Supplementary Code S1**.

### 2.6 Model evaluation and performance metrics

A comprehensive set of evaluation metrics was employed, including accuracy, precision, recall and F1-score. These metrics provide a balanced view of classifier performance, particularly in datasets with class imbalance, as they capture both sensitivity and specificity of the predictions. The confusion matrix was also utilized as an intuitive and informative tool for summarizing classification outcomes across true positives, true negatives, false positives, and false negatives. Additionally, the Receiver Operating Characteristic (ROC) curve was used to visualize the trade-off between the true positive rate and the false positive rate at various decision thresholds. The area under the ROC curve metric, derived from this curve, was used to quantify the model’s ability to distinguish between classes.

All metrics were averaged over multiple runs using Repeated Stratified k-Fold Cross-Validation. This method maintains the class distribution within each fold and reduces variance due to random sampling. Two different k-fold strategies were applied depending on the class distribution:

i) For the classification of paradormancy, ecodormancy and endodormancy, where the minority class had only 9 instances (paradormancy), a 3-fold repeated stratified cross-validation was used to ensure that each fold contained at least one instance from each class.

ii) For the classification of endormancy and ecodormancy, where the smallest class had 26 samples (ecodormancy), a 10-fold repeated stratified cross-validation was performed.

### 2.7 DNA methylation quantification, feature annotation, and visualization

WGBS data was processed to quantify cytosine methylation levels. Alignment metrics were parsed in R to calculate absolute and relative methylation levels across CpG, CHG, and CHH contexts. Absolute methylation percentages were computed per sample based on the ratio of methylated to total cytosines. For relative methylation, only methylated cytosines were considered, and their context-specific contributions were normalized to derive proportions, which were visualized per sample and chilling hour accumulation. Relevant cytosines were further classified by dormancy model (2-stages and 3-stages) and feature type (cytosine or region) and formatted into BED. Feature widths were calculated and their distributions compared globally and per model using histograms. Then we looked for shared and unique features across models. Features selected from the models were then classified into their genomic contexts: promoter (defined as 2kb upstream from the TSS), gene bodies, downstream region (2kb downstream from the transcription termination site), intergenic region (all locations different from promoter, 5′-UTR, exon, intron, 3′-UTR, or downstream), and a transposable elements (TEs) annotation developed by our group.

### 2.8 *P. avium* Tieton TEs annotation and TE-features downstream analysis

In order to identify TEs in *P. avium* Tieton, a *de novo* annotation using the RepeatModeler v2.0.5 tool was performed (Flynn et al., 2020). Since we lacked a reliable and curated database to search for specific transposable elements in *P. avium*. Using the BuildDatabase module, an index to use as RepeatModeler input was generated. This process writes a classification file that is then used as input for RepeatMasker v4.1.5 (Smit et al., 2019) and outputs a detailed annotation of identified transposable elements. Then the distribution of these TEs was characterized, since many features colocalized with TEs. Package BEDTools was used to intersect features from the classification models with genomic contexts: promoters, gene bodies, downstream region, intergenic region, and the TE annotation previously developed by our group. TE-associated to model relevant features were summarized by class and genomic context and visualized to identify context-specific enrichment patterns. All analyses and visualizations were performed in R. A circo plot showing this was made using the R packages: circlize, GenomicRanges, rtracklayer, and Biostrings; *P. avium* Tieton annotation file, and our TE annotation. Then, filtered by long terminal repeats (LTR) and splitted by chromosomes in 100 kb windows were added tracks of: LTR/ty3-retrotransposons, LTR/Copia, and the sum of both.

### 2.9 Features colocalization with quantitative trait loci

The distribution of methylated cytosines and regions on chromosome 4 was analyzed since several studies have identified QTLs associated with dormancy-related traits in sweet cherry. For this, four QTLs that were previously developed and associated with chilling requirements (CR), heat requirements (HR), flowering date (FD) (Castède et al., 2014), and fruit maturity date (MD) (Calle and Wünsch, 2020) were used.

## 3 Results

### 3.1 Whole-genome bisulfite sequencing of cultivars of *P. avium*

By understanding variations in DNA methylation and the context where methylated cytosine belongs, the regulatory roles of methylation in the genome can be discovered. The pattern and abundance of each mC-context contribute to the regulation of gene expression, transposon repression, or adaptation to environmental changes, among other functions in plants. The relative contribution of the three main DNA methylation contexts in sweet cherry cultivars and sampling years (**Supplementary Figure S1**) shows that across all samples, mCG consistently represents the largest proportion of methylated cytosines, with values ranging from 40% to 51%. Followed by the mCHG context, whose proportions are typically between 26% and 34%, while mCHH constitutes the smallest fraction, with values ranging from 21% to 32%. Overall, while the relative contributions of each methylation context are broadly similar across cultivars and years, subtle differences are evident among samples and conditions. Notably, samples from Experiment 3 Regina (Entre Ríos, 2022) (**Supplementary Figure S1**) present the highest mCG and lowest mCHH proportions. Across all cultivars, mCHG values remain intermediate and relatively stable. Additionally, absolute methylation levels were computed in each sample (**Supplementary Table S3**). CpG context is highly methylated in most of the samples. Experiment 1 (Kordia and Royal Dawn, 2015) showed a particularly high mCHG context, in both cultivars subject of study.

### 3.2 Feature subset selection

Feature selection was conducted independently for each input data type (cytosine-associated methylation and region-specific methylation) and under two classification scenarios: one including all three dormancy stages (3-stages: paradormancy, endodormancy, ecodormancy), and another using only endodormancy and ecodormancy samples (2-stages). In the three-class scenario, the cytosine dataset was reduced from 716,255 to 64 features, and the region-based dataset from 69,398 to 217 features. For the two-class setting, dimensionality was reduced to 297 and 535 features for the cytosine and regional datasets, respectively.

**Figure 2:**
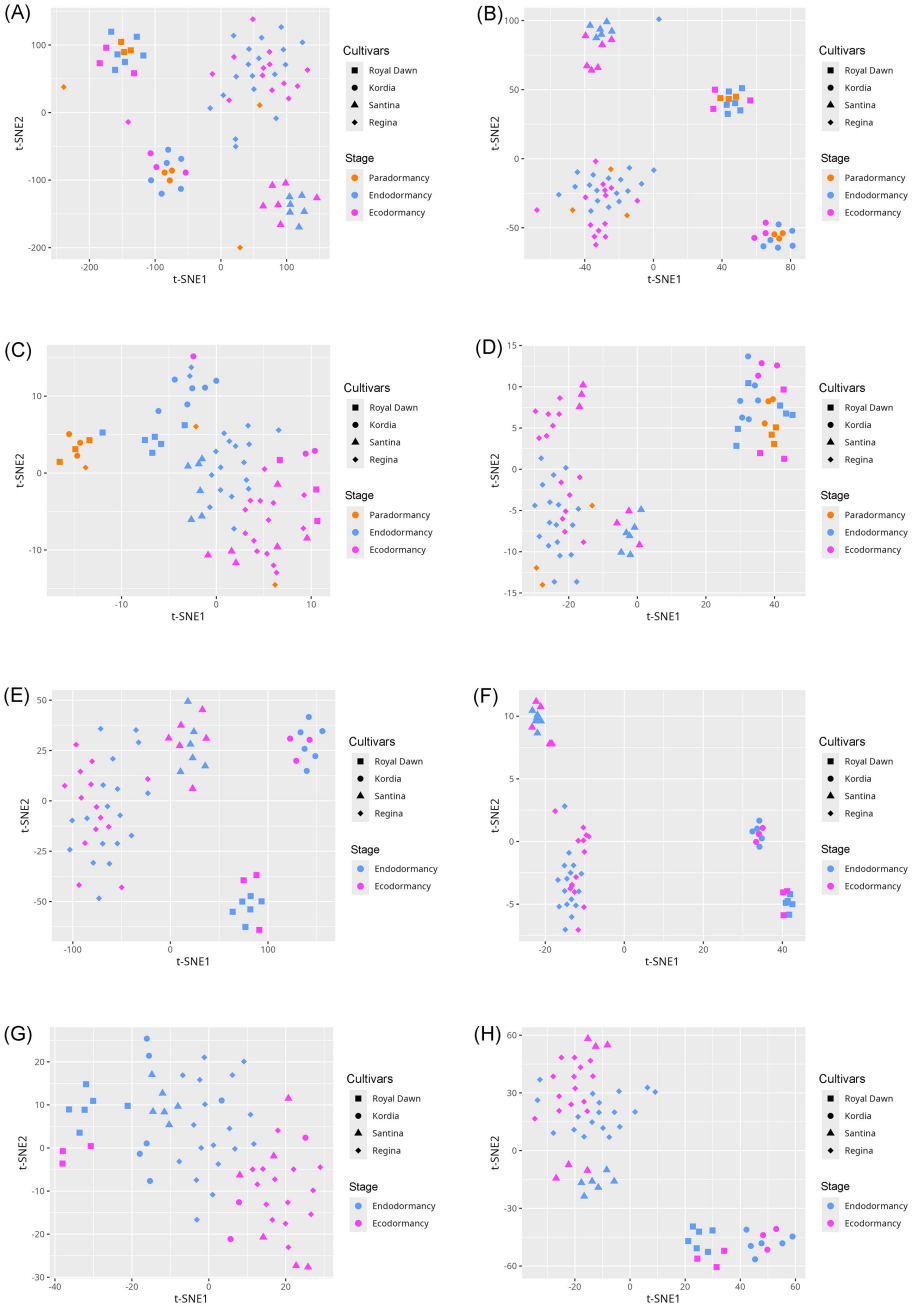
t-SNE visualization of cytosine- and region-level methylation profiles before and after feature selection across different model configurations. **(A, B)** Visualizations using the full feature sets in the three-stage model: cytosines **(A)** and methylated regions **(B)**. **(C, D)** Visualizations using only the informative features in the three-stage model: cytosines **(C)** and methylated regions **(D)**. **(E, F)** Visualizations using the full feature sets in the two-stage model: cytosines **(E)** and methylated regions **(F)**. **(G, H)** Visualizations using only the informative features in the two-stage model: cytosines **(G)** and methylated regions **(H)**.

To assess the impact of feature selection on the underlying data structure, we conducted a t-SNE analysis using both the full and the reduced feature sets. The initial analysis with all features primarily revealed sample clustering based on varietal identity across all comparisons, including both three-stage and two-stage (**Figure 2**) analyses of cytosine and regional methylation profiles. In contrast, when the analysis was limited to the subset of selected informative features, the resulting t-SNE plots demonstrated a clearer separation of samples according to dormancy stages (**Figure 2**).

### 3.3 Classification results

#### 3.3.1 Three dormancy stages: paradormancy, endodormancy and ecodormancy

Four distinct methylation datasets were analyzed: two cytosine-level datasets (all features: 716,255; informative features: 64) and two region-level datasets (all features: 69,398; informative features: 217). All datasets were evaluated separately using RF and XGB classifiers to classify samples into three dormancy stages: Ecodormancy, Endodormancy and Paradormancy.

**Figure 3:**
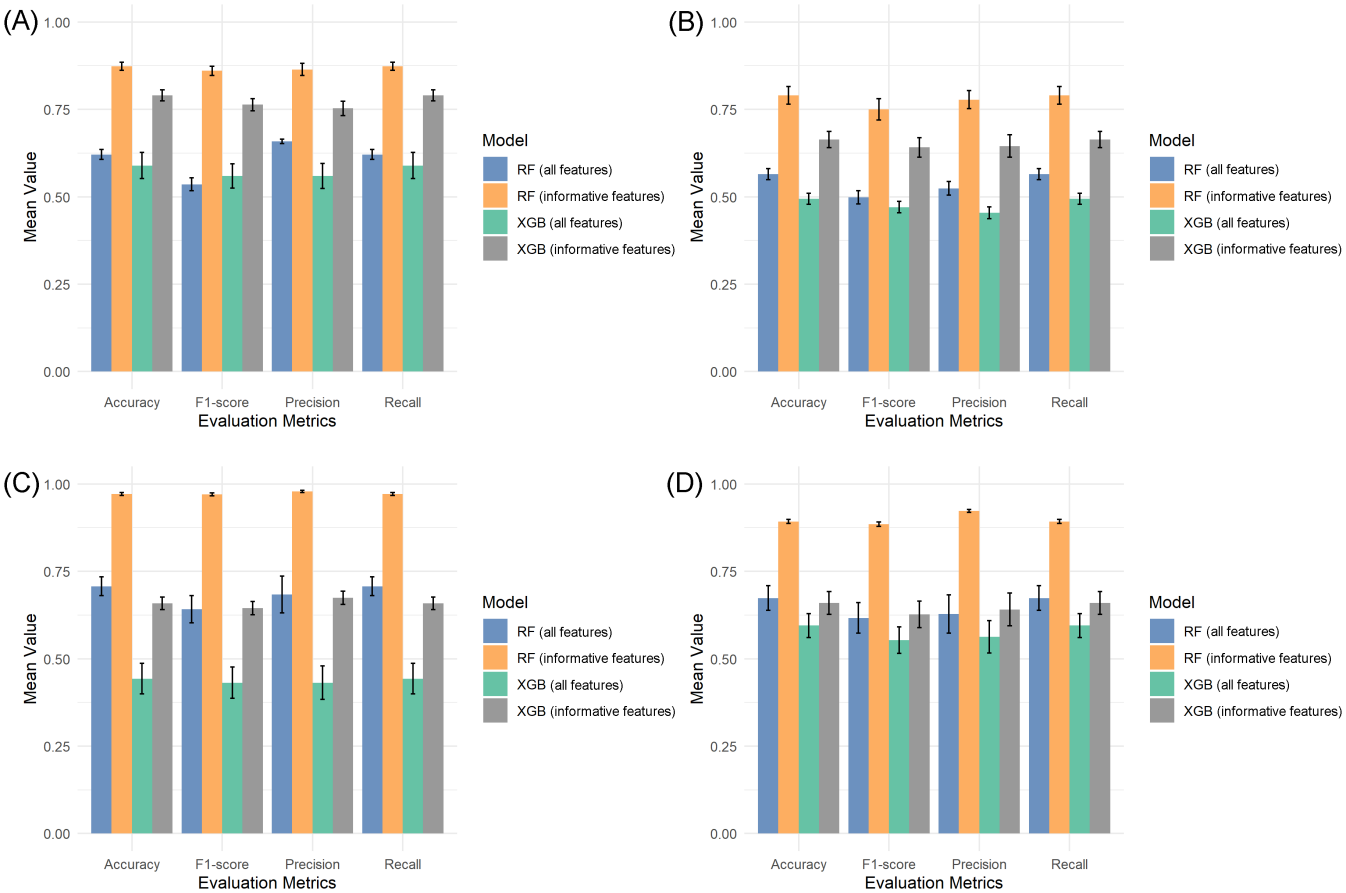
Performance comparison of RF and XGB models using full features and the informative feature dataset. **(A, B)** Model performances using cytosine-level data **(A)** and methylation region-level data **(B)** in the three-stage classification approach. **(C, D)** Model performances using cytosine-level data **(C)** and methylation region-level data **(D)** in the two-stage classification approach.

For the cytosine complete (unfiltered) dataset, RF achieved 62.1% accuracy, while XGB reached 59.0%. Similarly, with the full region dataset, RF attained 56.5% accuracy versus XGB’s 49.3% (**Figure 3**). Subsequent analysis using feature-selected datasets revealed significant improvements in classification performance. RF consistently outperformed XGB across all metrics (accuracy, precision, recall, F1-score). With the filtered cytosine dataset (64 informative features), RF achieved 87.3% mean accuracy compared to XGB’s 79.1%. The pattern held for the region dataset (217 features), where RF reached 79.1% accuracy versus XGB’s 66.4% (**Figure 3**). The t-tests confirmed that the performance differences between using all features and selected features were statistically significant (p< 0.05) for both classification models.

Paradormancy remained the most challenging class to predict in all scenarios. Confusion matrices (**Supplementary Figures S2** and **S3**) revealed that both RF and XGB consistently misclassified this class, especially when using the unfiltered datasets. In contrast, classification performance for endodormancy and ecodormancy improved after feature selection, with precision and recall values increasing for both models. The ROC curve analyses (**Supplementary Figure S4**) corroborated these findings. The RF model exhibited higher AUC values than XGB across both datasets with all features (cytosines: 0.73, regions: 0.72 for RF; cytosines: 0.73, regions: 0.70 for XGB) and for informative features (cytosines: 0.96, regions: 0.92 for RF; cytosines: 0.92, regions: 0.84 for XGB), further supporting the superiority of RF and the benefit of dimensionality reduction via feature selection.

#### 3.3.2 Two dormancy stages: endodormancy and ecodormancy

Due to the limited number of samples in the paradormancy class, which negatively impacted classification performance, this category was excluded from subsequent analyses. The focus of this stage was thus narrowed to the binary classification between endodormancy and ecodormancy stages, using both the cytosine-level and region-level methylation datasets.

The classification using the complete datasets exhibited low performance. Specifically, RF achieved an accuracy of 70.7% for cytosines and 67.4% for regions, while XGB reached only 44.3% and 59.5%, respectively. Confusion matrices from this analysis (**Supplementary Figure S5**, **S6**) revealed considerable misclassification across both models, and ROC analysis confirmed limited discriminative power in both datasets when no feature selection was applied. Classification performance improved markedly following the dimensionality reduction. As shown in (**Figures 3C, D**), RF achieved a mean accuracy of 97.1% for cytosines and 89.3% for regions, significantly outperforming XGB, which reached only 65.8% and 66.0%, respectively. Evaluation metrics (precision, recall, F1-score) mirrored this trend, and ROC analysis further demonstrated the superior discriminative ability of RF, which attained an AUC of 1.00 in the cytosine dataset and 0.99 in the region-based dataset, compared to 0.68 and 0.67 for XGB (**Supplementary Figure S7**). Moreover, the confusion matrices (**Supplementary Figures S5**, **S6**) illustrate these improvements, in the cytosine dataset, RF misclassified only three ecodormancy samples, whereas XGB misclassified 22 (7 of endodormancy and 15 of ecodormancy). For the regional data, RF made six errors, while XGB misclassified 21 samples in total (8 of endodormancy and 13 of ecodormancy).

These trends were further examined using t-SNE visualizations (**Figure 2**). When applied to the unfiltered datasets, samples tended to cluster primarily according to varietal background rather than dormancy stage. In contrast, the filtered datasets containing the selected informative features revealed more distinct groupings aligned with dormancy stages, although some degree of sample mixing remained. This residual overlap may stem from the inherent limitations of t-SNE, which emphasizes local rather than global structure. However, it is important to note that t-SNE was used solely for visualization purposes and played no role in classification model training.

#### 3.3.3 Explaining the model

Given the superior classification performance of the RF algorithm over XGB, SHAP analysis was applied to the RF models to interpret the contribution of individual features toward dormancy stage classification. This interpretability analysis focused exclusively on the informative feature sets for both the cytosine-level and region-level methylation datasets, as previously described.

**Figure 4:**
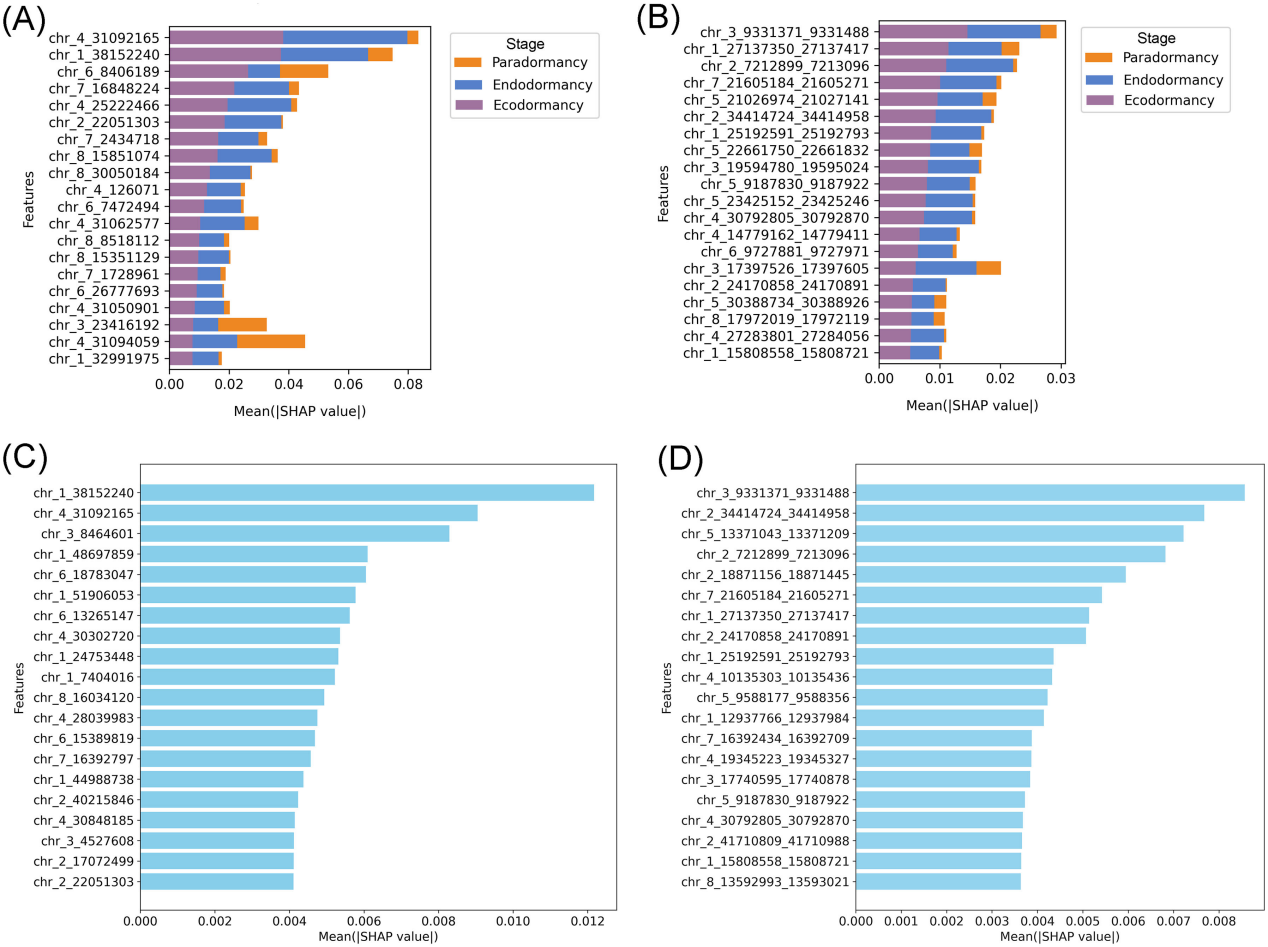
SHAP-based interpretation of feature contributions to dormancy stage classification. **(A, B)** SHAP summary plots showing the top 20 most impactful features for classification, based on **(A)** methylated cytosines and **(B)** methylated regions in the three-stage classification approach. **(C, D)** Global feature importance ranked by mean SHAP values for all samples, highlighting the features with the highest overall contribution using **(C)** cytosine-level and **(D)** region-level methylation data in the two-stage classification approach.

**Figures 4A, B** illustrates the SHAP summary plots corresponding to the three dormancy stages across both datasets. These visualizations aggregate SHAP values across all samples, providing a ranked overview of feature importance based on their average impact on the model’s predictions. In the cytosine dataset (**Figure 4A**), the features chr_4_31092165 (cytosine 31092165 located on chromosome 4) and chr_1_38152240 showed the highest overall impact, particularly in distinguishing the ecodormancy stage, as indicated by their strong SHAP contributions. Additional features such as chr_6_8406189, chr_7_16848224, and chr_4_25227466 also played relevant roles, with varying contributions across the three stages. Interestingly, certain features like chr_4_31050901 and chr_3_23416192 displayed more influence in differentiating paradormancy, suggesting stage-specific relevance. On the other hand, in the regional methylation dataset (**Figure 4B**), top features included chr_3_9331371_9331488 (region situated on chromosome 3, specifically among base pairs 9331371 to 9331488) and chr_1_27137350_27137417, both of which had a particularly strong association with the ecodormancy stage. Several other regions, such as chr_2_7212899_7213096 and chr_7_21605184_21605271, also contributed notably, with more nuanced effects across endodormancy and paradormancy. Notably, some regions, such as chr_6_9727681_9727971 and chr_3_31797583_31797605, exhibited relatively balanced contributions across all three dormancy stages, indicating potential roles in general dormancy regulation. Additionally, **Figures 4C, D** also illustrates the comparative feature importance analysis between two dormancy stages (endodormancy and ecodormancy). In the cytosine dataset (**Figure 4C**), features such as chr_4_31092165 and chr_1_38152240 stood out for their strong influence on the model’s predictions, particularly in distinguishing the ecodormancy stage. Other relevant markers included chr_6_8406189 and chr_4_25227466, which showed moderate contributions across both dormancy classes. Meanwhile, the methylation dataset (**Figure 4D**) highlighted chr_2_7212899_7213096 and chr_3_9331371_9331488 as the most impactful features, especially associated with ecodormancy. Some regions, like chr_6_9727681_9727971, exhibited more balanced SHAP values, suggesting shared roles in dormancy regulation regardless of the specific stage.

To complement the global analysis, individual SHAP bar plots were generated to explore how specific features contributed to the classification of correctly and incorrectly predicted ecodormancy samples. In **Supplementary Figure S8A**, the example shows an ecodormancy sample that was correctly classified. Most SHAP values are positive and substantial, particularly for features like chr_1_38152240 and chr_4_31092165, indicating strong support for the ecodormancy prediction. In contrast, **Supplementary Figure S8B** shows an ecodormancy sample that was misclassified as endodormancy by the model. Here, SHAP values for key features either decrease in magnitude or switch direction (e.g., chr_1_38152240 and chr_4_31092165 show negative contributions), effectively shifting the overall model output toward the wrong class. This shift in the SHAP value distribution in this sample highlights how changes in individual feature contributions can drive classification errors.scovery of genomic regions underlying dormancy stages.

**Figure 5:**
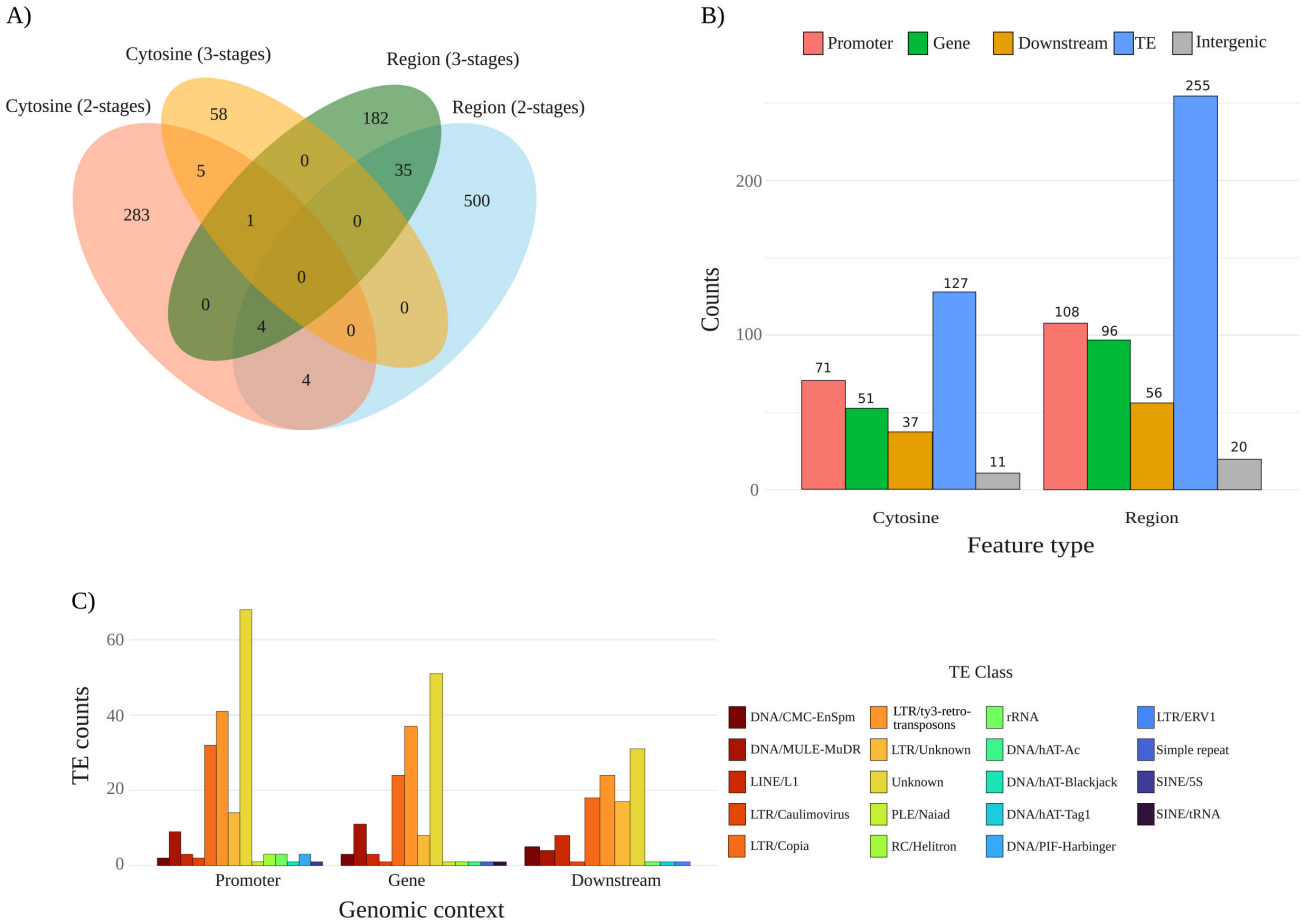
Features statistics and annotation. **(A)** Venn diagram of relevant features across the two models under study. Colored ellipses display the number of features per set: cytosine 2-stages (red), region 2-stages (light blue), cytosine 3-stages (orange), and region 3-stages (green). Overlaps are also indicated, displaying the number of either cytosines or regions shared, or as unique features per set. **(B)** Genomic annotation of features from the 2-stages model. Cytosine-level and region-level features were detected in the model in five genomic contexts: promoter (2 kb upstream of TSS), gene bodies (exonic and intronic regions), downstream (2 kb downstream 3’), transposable element (TE), and intergenic space (regions not overlapping annotated gene-proximal features). **(C)** Class-wise distribution of features from model 2-stages overlapping TEs and their genomic context (Promoter, Gene, and Downstream). Colored bars depicting TE classes based on our curated annotation.

To understand the common and unique features of each model, including the overlap of individual cytosines within regions, pairwise and multi-set intersection distribution of the relevant features detected by the two models are shown in **Figure 5A**. The largest unique subset corresponds to region features from the 2-stages model with 500 regions, followed by 283 unique cytosines. The 3-stages model contributes with 182 unique regions and 58 unique cytosines. Among shared features, only 35 regions and 5 cytosines are common to both models. Relatively few individual cytosines overlap regions, four cytosines in the 2-stages model co-localize with regions from the same model, and another four 2-stages cytosines overlap regions from both models. A single cytosine is shared by the two models and is located within a region feature from the 3-stages model. In addition, no cytosine is common to all four datasets. The 2-stages model yields the largest feature sets and the greatest number of model-unique features.

In order to understand the potential functional roles of relevant features, we looked at their genomic context (**Figure 5B**. See **Supplementary Figure S9** for the 3-stages model). From a total of 297 cytosines and 535 regions relevant for the 2-stages model, many of the features are found in transposable elements (TEs), with 127 cytosines and 255 regions located in TEs. Then, the following most frequent context is promoters, with 71 cytosines and 108 regions, followed by gene bodies, which harbor 51 cytosines and 96 regions. In the downstream context, we found 37 cytosines and 56 regions, whereas intergenic space contributes the fewest number of features, harboring 11 cytosines and 20 regions. In every context, region-level counts exceed cytosine-level counts.

Our pipeline identified a total of 52.79% transposable elements (TE) in the *P. avium* cv. Tieton genome. With that curated annotation, TEs with features were classified by genomic context and class (**Figure 5C**. See **Supplementary Figure S10** for 3-stages model). From our TE annotation, 19 classes (including unknown) were found distributed among promoters, gene bodies, and downstream regions. Even though features from the 2-stages model were found in intergenic regions, none of the TEs with features are located in that genomic context (**Supplementary Table S4**). Regarding TE classes, and besides unknown elements, two classes were enriched in each of the genomic contexts: LTR/ty3-retrotransposons, and LTR/Copia. Overall, the data indicates a consistent enrichment of LTR-type across regulatory (promoter), coding (gene), and proximal downstream segments, with fewer contributions from other TE classes. LTR/ty3-retrotransposons elements cluster toward pericentromeric or proximal regions of several chromosomes, most conspicuously on chromosomes 1, 4, and 5 (**Supplementary Figure S11**). LTR/Copia TEs, by contrast, are more dispersed along chromosomal arms. Conversely, chromosomes 3, 6, 7, and 8 exhibit comparatively sparse LTR coverage.

**Figure 6:**
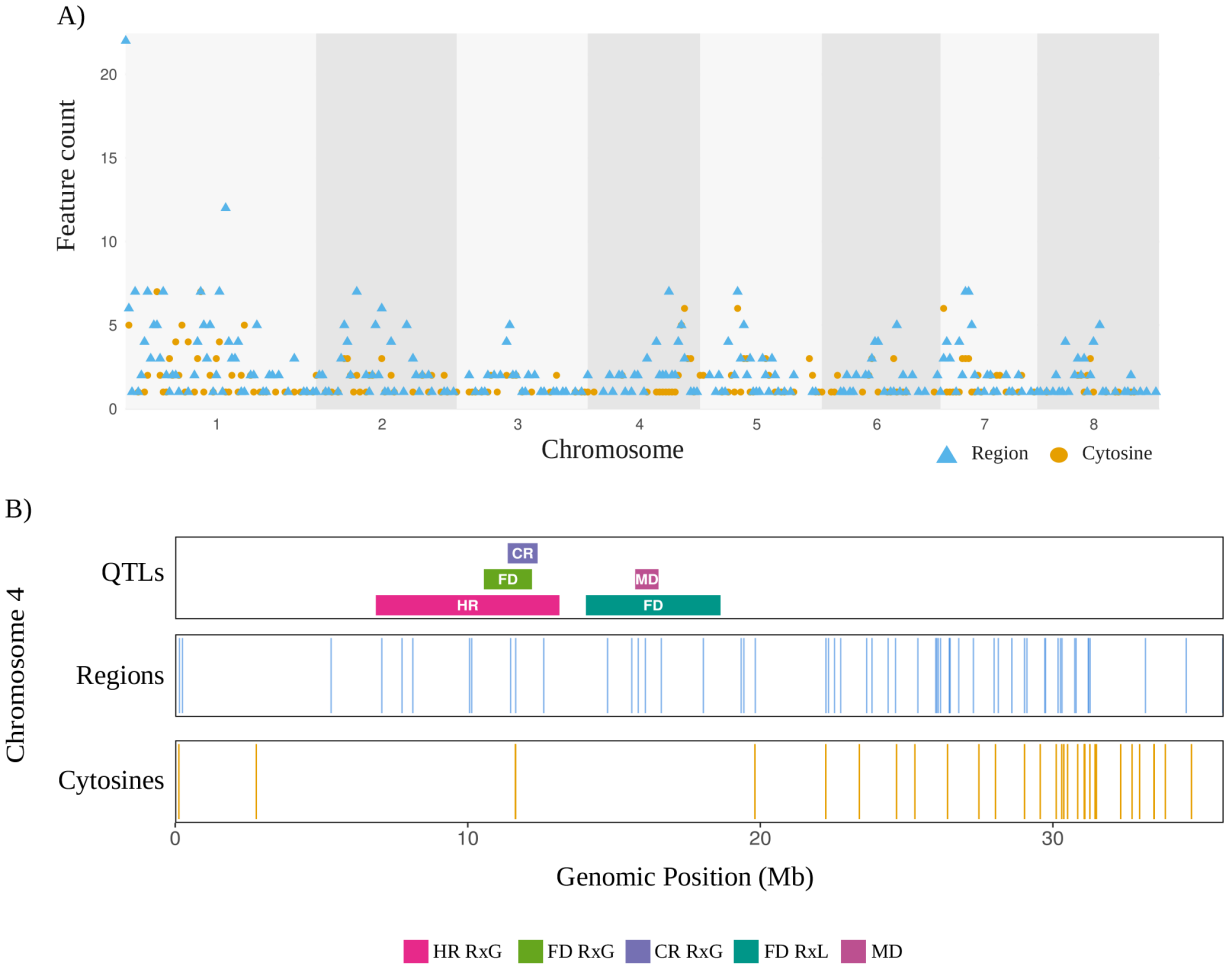
Genomic distribution of features from the 2-stages model and associated QTLs. **(A)** The Manhattan plot displays the density of features across the eight chromosomes of P. avium, binned in 1 Mb intervals. The distribution of cytosine-level features is shown as orange dots, representing the counts of individual cytosines per 1 Mb window. Region-level features are depicted as blue triangles, indicating the number of regions per 1 Mb window. The corresponding chromosomes and genomic positions are indicated on the X axis as megabases (Mb). **(B)** Genomic distribution of features along P. avium chromosome 4. Each track position is based on the Tieton v2.0 genome annotation, in megabases. QTLs associated with flowering date (FL), chilling requirements (CR), and heat requirements (HR), in either RxG (Regina x Garnet) or RxL (Regina x Lapins) progenies were reported by Castède et al. (2015); and maturity date (MD) reported by Calle and Wünsch (2020). Blue lines indicating region features, and orange lines indicating single cytosine features. Cytosines and regions shorter than 20 kb were expanded to this width for improved visualization.

Features from the model were later visualized to address their distribution along the eight chromosomes of *Prunus avium* (**Figure 6A**). Chromosome 1 exhibits the highest overall feature density. A thorough examination of the chromosome reveals an abundance of region counts, with some regions exhibiting values exceeding 20 megabase (Mb). This observation is further complemented by the presence of a significant number of cytosines. Chromosomes 3, 6, and 7 exhibit low overall region densities. The distribution of chromosome 4 features counts primarily located within the range of 25 to 32 Mb, and exhibiting windows that harbor both feature types. Chromosome 5 exhibits a distinctive mid-chromosomal high density at 10–15 Mb, with a high number of cytosine counts, and several regions. Chromosome 8 exhibits a limited number of windows with regions and reduced cytosine densities. The colocalization of cytosines and regions in high-density within the chromosomes can be observed, including chromosome 1 at 0–2 Mb and chromosome 5 at 10–15 Mb. Overall, features are distributed along the genome with some cytosines clustering within genomic regions of interest such as previously reported QTLs in *P. avium*. **Figure 6B** shows the colocalization between model features and QTLs associated with flowering date, temperature requirements (chill and heat), and maturity date. These QTLs were all identified in chromosome 4 of *P. avium* cv. Tieton, highlighting the relevance of linkage group 4 by carrying several QTLs associated with dormancy related traits.

## 4 Discussion

The accurate and timely assessment of dormancy stages is a critical challenge in perennial horticulture, directly impacting orchard management and yield optimization. In this study, high-resolution DNA methylation profiling and machine learning techniques were integrated to identify robust epigenetic markers for predicting dormancy in sweet cherry. In plants, methylation occurs in three different contexts that serve a variety of roles in mediating environmental signals with molecular regulation. mCG methylation is found throughout the genome, including gene bodies, promoters, and repetitive regions, while mCHG and mCHH methylation are particularly enriched in repetitive sequences, including transposable elements, and play a key role in silencing these elements to maintain genome stability.

The results showed that in the three-class scenario, when methylated cytosines data were used, the paradormancy stage was misclassified 100% of the time by the RF model and 78% of the time by XGBoost. In contrast, endodormancy and ecodormancy had misclassification rates of 0% and 31% with RF, and 25% and 50% with XGBoost, respectively. Similar results were observed in methylated regions, where paradormancy exhibited lower classification efficiency compared to endodormancy and ecodormancy. This pattern may be explained by the imbalance in the number of samples per class, which appears to correlate with the error rate, with paradormancy having the fewest samples (9) and endodormancy having the most (36). Due to this class imbalance, both models tended to misclassify paradormancy samples, often assigning them to the majority class, endodormancy. The poor predictive performance in classifying unbalanced datasets can be since machine learning algorithms are designed to maximize overall accuracy and may struggle to accurately classify the minority class (Imani et al., 2025). The results of this study showed that RF outperformed XGBoost in accuracy and efficiency across all classes, regardless of class imbalance. Similar findings were reported by Dube and Verster (2023), who observed that RF demonstrated robustness to class imbalance and outperformed several machine learning models, including naïve Bayes, XGBoost, and k-nearest neighbors, which struggled with imbalanced data.

Feature selection applied to methylated cytosines and regions improved classification accuracy by approximately 40% for RF and 34% for XGBoost, highlighting the effectiveness of this approach. Similar findings were reported by Hassan et al. (2023), where feature selection improved the accuracy of all classification models. This improvement is due to the effective selection of features, which ensures that the most relevant information is extracted from the dataset, enabling more accurate classification (Javidan et al., 2024). Bolón-Canedo and Alonso-Betanzos (2019) pointed out that different feature selection methods can yield varying subsets from the same dataset; thus, the results may lack stability. Therefore, combining multiple feature selection techniques, rather than relying on a single method, helps control variance, mitigates the limitations of individual approaches, and offers a more comprehensive view of feature relevance, which makes this approach successful (Bolón-Canedo and Alonso-Betanzos, 2019). Therefore, the approach presented in this study combines three algorithms for identifying relevant variables, since the combination provides a better approximation based on the idea that “two heads are better than one” (Bolón-Canedo and Alonso-Betanzos, 2019). Consistent with the classification results, RF outperformed XGBoost in accuracy following feature selection. Additionally, using methylated cytosine data proved more effective than using methylated regions, improving classification accuracy by 11% in RF and 19% in XGBoost. Similarly, Hassan et al. (2023) also showed that RF consistently outperformed XGBoost, both on the full dataset and after feature selection. Notably, the improvement in the classification after feature selection was also reflected in the t-SNE plots, since the clustering with the original data set, samples tended to cluster by cultivar rather than dormancy stage, whereas after feature selection, the clustering aligned more clearly with dormancy stages.

Despite the overall increase in accuracy after feature selection, the classification error for paradormancy samples remained high, ranging from 33% (with methylated cytosines using RF) to 78% (with methylated regions using XGBoost). This highlights a significant challenge in accurately classifying the paradormancy stage compared to endodormancy and ecodormancy. These results suggest that neither RF nor XGBoost was able to overcome the issue of class imbalance, even after applying feature selection. It is worth noting that the SMOTE algorithm, which is designed to generate synthetic examples of the minority class and alleviate class imbalance (Fernández et al., 2018), was also tested (data not shown). However, its application did not lead to improved classification performance. As a result, in the second scenario, paradormancy was excluded from the dataset, and only endodormancy and ecodormancy were used to train and build the classification models.

In the second scenario, where only endodormancy and ecodormancy were considered, the RF model showed an increase in accuracy of 15% with methylated cytosines and 9% with methylated regions, compared to the three-class scenario. In contrast, XGBoost experienced a decline in accuracy of 24% and 18% for cytosines and regions, respectively. Consistent with the second scenario observations, both models showed improved accuracy when feature selection was applied, reinforcing the importance of identifying the most informative variables. However, it is important to note that the observed increase in accuracy does not necessarily reflect an improvement in the overall classification performance, since in the three-class scenario, much of the accuracy lost was tied to the misclassification of paradormancy samples. In both scenarios, RF consistently achieved the highest accuracy across all configurations, confirming its superior performance and greater stability under both balanced and imbalanced conditions. These findings further emphasize the limitations of XGBoost when dealing with skewed datasets, as well as the benefit of applying feature selection to enhance the model performance of dormancy stages classification based on epigenetic information.

Given that RF consistently outperformed XGBoost in accuracy and robustness across all scenarios, interpretability efforts based on SHAP focused on RF models trained with the informative feature sets of both cytosine-level and region-level methylation data. In the cytosine dataset, features such as chr_4_31092165 and chr_1_38152240 emerged as the most impactful, particularly for distinguishing the ecodormancy stage. This suggests that methylation at these loci could be tightly associated with epigenetic regulation mechanisms unique to ecodormancy. Other features, like chr_3_23416192 and chr_4_31050901, displayed stronger relevance for paradormancy, indicating that although paradormancy was difficult to classify due to data imbalance, certain epigenetic markers do exist that could potentially improve its prediction if more balanced data were available. Similarly, in the regional methylation dataset, regions like chr_3_9331371_9331488 and chr_1_27137350_27137417 had high SHAP values. Interestingly, some regions, such as chr_6_9727681_9727971, demonstrated relatively balanced contributions across all three dormancy stages. This may indicate shared or transitional roles in the broader regulation of dormancy, potentially pointing to core epigenetic signatures common to different dormancy stages. The individual SHAP plots for each dormancy stage (**Supplementary Figure S8**) showed that features such as chr_1_38152240 contributed strongly and positively to the correct classification of ecodormancy samples, while exhibiting negative contributions in misclassified samples labeled as endodormancy. This highlights the high discriminative power of this feature in separating these two stages. In contrast, features like chr_3_8464601, although showing high SHAP values, presented similar contributions in both ecodormancy and endodormancy samples. This suggests that it may not be informative on its own but could contribute to the model’s predictions through interactions with other features, an effect often captured by ensemble models like SHAP (Štrumbelj and Kononenko, 2014).

There are several examples in fruit trees where methylation of individual cytosines plays key roles during dormancy. Kumar et al. (2016) examined four developmental stages in apple, including dormant buds, and showed, using bisulfite–sequenced MSAP fragments, that the number of methylated cytosines at single loci correlated with transcript levels of those genes (e.g., an acid phosphatase 1-like gene and a galactose oxidase-like gene). Prudencio et al. (2018) in almond found that over 90 % of cytosines are unmethylated, while approximately 1-1.3 % are fully methylated in CpG contexts; these polymorphic 5-mC sites were conserved across two years and between dormant and non–dormant buds. These studies demonstrate that methylation at even a handful of cytosines in specific genomic contexts can meaningfully modulate gene expression. Given their stability and stage specificity, single–cytosine marks hold considerable promise as robust biomarkers for dormancy transitions. Although the precise mechanism by which a single methylated cytosine influences gene expression remains unclear, we hypothesize that such effects occur when the cytosine resides within functionally relevant regions, particularly transcription factor binding sites or cis–regulatory modules.

Differences in the number of relevant features were evident between the dormancy models. The 2-stages model generated the largest and most unique feature sets, resulting in 500 regions and 283 cytosine unique features. This genome-wide representation is consistent with the higher predictive power of 2-stage cytosine methylation data in Random Forest and XGBoost, suggesting that the additional loci due to the higher input samples, supplies informative data that improves the classification. By contrast, the 3-stage model contributed a leaner, yet distinct, repertoire of 182 unique regions and 58 unique cytosines, which may represent the early dormancy stage paradormancy signatures that the 2-stage model misses.

The reduced number of individual cytosine overlapping regions in the 2-stages model (4) may imply that single cytosines show more sensitivity to coverage noise or local sequence context, whereas regions provide a more robust, though less precise, relevant dormancy-associated loci. In order to address those questions, we searched for the genomic context of those relevant features from the 2-stage model to gather insights about the functional roles of those cytosines (**Figure 5B**). The genomic context frequencies rank in the same order across both feature types. From highest to lowest: TEs, promoter, gene bodies, downstream, and intergenic, indicating that cytosine and region features share similar genomic preferences. Since TEs were the highest genomic context with features, and they can be located in any of the above genomic contexts, we further study the genomic context of TEs with features and their corresponding class (**Figure 5**). The number of TEs that remained unclassified is still high for *P. avium* Tieton annotation, highlighting the need for cultivar-specific transposable elements annotation.

Certain TE families exhibit preference for specific genomic features. A well known example is ONSEN, an LTR/Copia element in *Arabidopsis thaliana* that integrates preferentially within genes (Merkulov et al., 2025). Members of the LTR/Copia class often insert near gene-rich regions, unlike other TEs that target intergenic areas. Several Copia families including Copia87/ONSEN (Ito et al., 2011), COPIA37, TERESTRA, and ROMANIAT5 (Pietzenuk et al., 2016) have been well described as stress-responsive TEs, becoming active when temperature rises due to heat-responsive elements in their promoter regions. The LTR/Copia and Ty3, flanked by a promoter region, can undergo a replication cycle (Oberlin et al., 2017), with the later ability to confer neighboring genes the responsiveness to abiotic stressors (Xu et al., 2024). LTR/ty3-retrotransposons elements in *P. avium* were found predominantly in heterochromatic regions and LTR/Copia elements located in euchromatic domains, consistent with observations in almond (Alioto et al., 2020). TE activity is often suppressed through epigenetic mechanisms such as the RNA-directed DNA methylation (RdDM) pathway, which is responsive to environmental conditions. Nozawa et al. (2022) reported that ONSEN expression varies across *Arabidopsis* ecotypes depending on DNA methylation levels, with the Kyoto ecotype showing increased expression due to reduced methylation conferring release from transcriptional silencing. Although the functional dynamics of TE regulation during dormancy remain unclear in sweet cherry, we hypothesize that small RNAs involved in RdDM, previously characterized in *P. avium* (Rothkegel et al., 2017; 2020; Kuhn et al., 2025), may regulate TE transcription and potentially modulate the expression of adjacent genes involved in dormancy transitions.

Mapping these loci along the eight chromosomes of *P. avium* (**Figure 6A**) revealed broad genome coverage, with particularly relevant regions on chromosome 1 (0–2 Mb), chromosome 4 (25–32 Mb), and chromosome 5 (10–15 Mb). Chromosome 4 (LG4, **Figure 6B**) includes a well reported QTL hotspot where flowering date (FD), chilling requirement (CR), and heat requirement (HR) QTLs, identified in sweet cherry progenies with Regina as the progenitor (Regina × Lapins and Regina × Garnet), co-localize with maturity date (MD) QTLs (Castède et al., 2014; Calle and Wünsch, 2020; Calle et al, 2020). Our model identified a concentration of informative regions, with fewer cytosine features, suggesting a broader chromatin-level regulation rather than single-site CpG variation may underlie dormancy transitions. This is consistent with previous reports demonstrating that DNA methylation and chromatin remodeling regulate dormancy release in *Prunus* (Kuhn et al., 2025; Rothkegel et al., 2020; Zhu et al., 2020). Within chromosome 4, several biologically relevant candidate genes have been reported (Castède et al., 2014; Calle and Wünsch, 2020). These include *EMF2*, a MADS-box transcription factor involved in floral meristem identity (Yoshida et al., 2001); *NUA* (Nuclear Pore Anchor), which regulates nuclear-cytoplasmic transport and chromatin organization; NAC domain transcription factors associated with stress responses and developmental control; *EXPA1* (Expansin A1), involved in cell wall loosening and bud outgrowth; bHLH (basic helix-loop-helix) transcription factors implicated in hormone and light signaling pathways; and WRKY transcription factors known for their role in stress-responsive gene regulation.

Additionally, a QTL on LG1 includes *AGL24*-like MADS-box genes, which are involved in floral transition and respond to environmental cues, like the *DORMANCY-ASSOCIATED MADS-box* (*DAM*) gene cluster (*DAM1* to *DAM6*) (Castède et al., 2015), which also overlaps with regions selected by our model. These genes have been shown to play a central role in the repression of bud growth during dormancy and its release in response to chilling accumulation. Castède et al. (2014) identified this region as a key determinant of phenological variation in sweet cherry, particularly in high-chill cultivars such as Regina. Additionally, Calle and Wünsch (2020) demonstrated that this QTL on LG1 remains significant even in low-chill cultivars, supporting its broad importance across diverse genetic backgrounds. In our study, several methylation features selected by the model colocalized with this QTL region, suggesting that epigenetic regulation contributes to differential *DAM* genes expression among cultivars. Increasing evidence supports the role of DNA methylation in modulating *DAM* genes activity during dormancy transitions. Zhu et al. (2020) showed that changes in methylation levels at the promoters and gene bodies of *DAM* genes in peach were associated with their transcriptional downregulation during chilling accumulation. Specifically, DNA hypomethylation correlated with a reduction in *DAM* transcript levels, suggesting that chilling-induced epigenetic remodeling facilitates dormancy release. Similar patterns of dynamic methylation have been reported in other *Prunus* species, underscoring the conserved nature of this regulatory mechanism. Our results align with these findings and point to DNA methylation as a key regulatory layer influencing the dormancy-related function of *DAM* genes. These observations support a model in which genetic loci like *DAM* are subject to cultivar-specific epigenetic modulation, contributing to phenotypic diversity in dormancy behavior across sweet cherry germplasm. Altogether, these results show the relevance of the LG1 QTL and the *DAM* cluster as conserved regulators of dormancy, while also highlighting the potential role of epigenetic variability in shaping cultivar-specific responses to chilling.

## 5 Conclusion

This study presents a new integrating frame that combines the high-resolution DNA methylation profile with automatic learning approaches to classify dormancy stages in *Prunus avium*. The results showed that the methylation data at the cytosine level, when processed through the selection of features and interpreted through form values, provide highly informative epigenetic markers to distinguish endodormancy and ecodormancy stages with high precision. Among the proven models, RF constantly exceeded XGBoost in robustness, precision, and interpretability in balanced and unbalanced scenarios. It is important to note that some of the methylation features co-localize with related QTLs previously identified by other groups, such as chilling and heat requirement flowering date and maturity date. The results of this study contribute a valuable methodological and conceptual advance for epigenetic research in these traits. It provides a fundamental resource for future functional validation studies and prepares the scenario to develop predictive tools in horticultural management. When discovering key methylation markers and their genomic contexts, this study improves our understanding of dormancy regulation and underlines the importance of epigenetic mechanisms in the adaptation and resistance of perennial crops.

## Data Availability

The original contributions presented in the study are included in the article/**Supplementary Material**. Further inquiries can be directed to the corresponding authors.

## References

[B1] Alioto T. Alexiou K. G. Bardil A. Barteri F. Castanera R. Cruz F.. (2020). Transposons played a major role in the diversification between the closely related almond and peach genomes: results from the almond genome sequence. Plant J. 101, 455–472. doi: 10.1111/tpj.14538, PMID: 31529539 PMC7004133

[B2] Alonso-González E. Gutmann E. Aalstad K. Fayad A. Gascoin S. (2020). Snowpack dynamics in the Lebanese mountains from quasi-dynamically downscaled ERA5 reanalysis updated by assimilating remotely-sensed fractional snow-covered area. Hydrol. Earth Syst. Sci. 25, 1–31. doi: 10.5194/hess-25-4455-2021

[B3] Andrews S. (2010). FastQC: A Quality Control Tool for High Throughput Sequence Data. Available online at: http://www.bioinformatics.babraham.ac.uk/projects/fastqc/ (Accessed September 20, 2024).

[B4] Baumgarten F. Zohner C. M. Gessler A. Vitasse Y. (2021). Chilled to be forced: The best dose to wake up buds from winter dormancy. New Phytol. 230, 1366–1377. doi: 10.1111/nph.17270, PMID: 33577087

[B5] Bi Y. Xiang D. Ge Z. Jia C. Song J. (2020). An interpretable prediction model for identifying N7-methylguanosine sites based on XGboost and SHAP. Mol. Ther. 22, 362–372. doi: 10.1016/j.omtn.2020.08.022, PMID: 33230441 PMC7533297

[B6] Bolón-Canedo V. Alonso-Betanzos A. (2019). Ensembles for feature selection: A review and future trends. Inf. Fusion 52, 1–12. doi: 10.1016/j.inffus.2018.11.008

[B7] Breiman L. (1996). Bagging predictors. Mach. Learn. 24, 123–140. doi: 10.1007/BF00058655

[B8] Bzdok D. Altman N. Martin K. (2018). Statistics versus machine learning. Nat. Methods 15, 233–234. doi: 10.1038/nmeth.4642, PMID: 30100822 PMC6082636

[B9] Calle A. Cai L. Iezzoni A. Wünsch A. (2020). Genetic dissection of bloom time in low chilling sweet cherry (*Prunus avium* L.) using a multi-family QTL approach. Front. Plant Sci. 10. doi: 10.3389/fpls.2019.01647, PMID: 31998337 PMC6962179

[B10] Calle A. Wünsch A. (2020). Multiple-jpopulation QTL mapping of maturity and fruit-quality traits reveals LG4 region as a breeding target in sweet cherry (*Prunus avium* L.). Hortic. Res. 7, 127. doi: 10.1038/s41438-020-00349-2, PMID: 32821410 PMC7395078

[B11] Castède S. Campoy J. A. Le Dantec L. Quero-Garcia J. Barreneche T. Wenden B.. (2015). Mapping of candidate genes involved in bud dormancy and flowering time in sweet cherry (*Prunus avium*). PloS One 10, e0143250. doi: 10.1371/journal.pone.0143250, PMID: 26587668 PMC4654497

[B12] Castède S. Campoy J. A. Quero-García J. Le Dantec L. Lafargue M. Barreneche T.. (2014). Genetic determinism of phenological traits highly affected by climate change in *Prunus avium*: flowering date dissected into chilling and heat requirements. New Phytol. 202, 703–715. doi: 10.1111/nph.12658, PMID: 24417538

[B13] Chen T. Guestrin C. (2016). “XGBoost: A scalable tree boosting system,” in KDD '16: Proceedings of the 22nd ACM SIGKDD International Conference on Knowledge Discovery and Data Mining. (New York, NY, United States: Association for Computing Machinery), 785–794. doi: 10.1145/2939672.2939785

[B14] Chu Y. R. Jo M. S. Kim G. E. Park C. H. Lee D. J. Che S. H.. (2024). Non-destructive seed viability assessment via multispectral imaging and stacking ensemble learning. Agriculture 14, 1679. doi: 10.3390/agriculture14101679

[B15] Cline M. G. Deppong D. O. (1999). The role of apical dominance in paradormancy of temperate woody plants: a reappraisal. J. Plant Physiol. 155, 350–356. doi: 10.1016/S0176-1617(99)80116-3

[B16] Considine M. J. Considine J. A. (2016). On the language and physiology of dormancy and quiescence in plants. J. Exp. Bot. 67, 3189–3203. doi: 10.1093/jxb/erw138, PMID: 27053719

[B17] Ding J. Wang K. Pandey S. Perales M. Allona I. Khan M. R. I.. (2024). Molecular advances in bud dormancy in trees. J. Exp. Bot. 75, 6063–6075. doi: 10.1093/jxb/erae183, PMID: 38650362 PMC11582002

[B18] Dube L. Verster T. (2023). Enhancing classification performance in imbalanced datasets: A comparative analysis of machine learning models. Data Sci. Finance Econom. 3, 354–379. doi: 10.3934/DSFE.2023021

[B19] Fadón E. Herrero M. Rodrigo J. (2015). Flower development in sweet cherry framed in the BBCH scale. Sci. Hortic. 192, 141–147. doi: 10.1016/j.scienta.2015.05.027

[B20] Fernández A. García S. Herrera F. Chawla N. V. (2018). SMOTE for Learning From Imbalanced Data: Progress and Challenges, Marking the 15-Year Anniversary. J. Artif. Intell. Res. 61, 863–905. doi: 10.1613/jair.1.11192

[B21] Flynn J. M. Hubley R. Goubert C. Rosen J. Clark A. G. Feschotte C.. (2020). RepeatModeler2 for automated genomic discovery of transposable element families. Proc. Natl. Acad. Sci. U. S. A. 117, 9451–9457. doi: 10.1073/pnas.1921046117, PMID: 32300014 PMC7196820

[B22] Hassan M. M. Hassan M. M. Yasmin F. Khan M. A. R. Zaman S. Islam K. K.. (2023). A comparative assessment of machine learning algorithms with the Least Absolute Shrinkage and Selection Operator for breast cancer detection and prediction. Decision Analytics J. 7, 100245. doi: 10.1016/j.dajour.2023.100245

[B23] Imani M. Beikmohammadi A. Arabnia H. R. (2025). Comprehensive analysis of random Forest and XGBoost performance with SMOTE, ADASYN, and GNUS under varying imbalance levels. Technologies 13, 88. doi: 10.3390/technologies13030088

[B24] Ito H. Gaubert H. Bucher E. Mirouze M. Vaillant I. Paszkowski J. (2011). An siRNA pathway prevents transgenerational retrotransposition in plants subjected to stress. Nature 472, 115–119. doi: 10.1038/nature09861, PMID: 21399627

[B25] Javidan S. M. Banakar A. Rahnama K. Vakilian K. A. Ampatzidis Y. (2024). Feature engineering to identify plant diseases using image processing and artificial intelligence: A comprehensive review. Smart Agric. Technol. 8, 100480. doi: 10.1016/J.ATECH.2024.100480

[B26] Kavzoglu T. Teke A. (2022). Predictive performances of ensemble machine learning algorithms in landslide susceptibility mapping using random forest, extreme gradient boosting (XGBoost) and natural gradient boosting (NGBoost). Arab. J. Sci. Eng. 47, 7367–7385. doi: 10.1007/s13369-022-06560-8

[B27] Krueger F. Andrews S. R. (2011). Bismark: a flexible aligner and methylation caller for Bisulfite-Seq applications. Bioinformatics 27, 1571–1572. doi: 10.1093/bioinformatics/btr167, PMID: 21493656 PMC3102221

[B28] Krueger F. James F. Ewels P. E. E A. Weinstein M. Schuster-Boeckler B. (2016). Felixkrueger/Trimgalore: V0.6.10. Available online at: https://github.com/Felixkrueger/Trimgalore (Accessed September 20, 2024).

[B29] Kuhn N. Arellano M. Ponce C. Hodar C. Correa F. Multari S.. (2025). RNA-seq and WGBS analyses during fruit ripening and in response to ABA in sweet cherry (*Prunus avium*) reveal genetic and epigenetic modulation of auxin and cytokinin genes. J. Plant Growth Regul. 44, 1165–1187. doi: 10.1007/s00344-024-11340-9

[B30] Kumar G. Rattan U. K. Singh A. K. (2016). Chilling-mediated DNA methylation changes during dormancy and its release reveal the importance of epigenetic regulation during winter dormancy in apple (*Malus* x *domestica* borkh.). PloS One 11, e0149934. doi: 10.1371/journal.pone.0149934, PMID: 26901339 PMC4763039

[B31] Li B. Xia X. Liu S. (2015). Changes in physiological and biochemical properties and variation in DNA methylation patterns during dormancy and dormancy release in blueberry (*Vaccinium corymbosum* L.). Plant Physiol. J. 51, 1133–1141.

[B32] Li Y. Li R. Ji R. Wu Y. Chen J. Wu M.. (2024). Research on factors affecting global grain legume yield based on explainable artificial intelligence. Agriculture 14, 438. doi: 10.3390/agriculture14030438

[B33] Liu Y. Wang Y. Zhang J. (2012). “New machine learning algorithm: Random forest,” in Information Computing and Applications: Third International Conference, ICICA 2012, Chengde, China, September 14-16, 2012. 246–252 (Berlin, Heidelberg: Springer).

[B34] Luedeling E. Girvetz E. H. Semenov M. A. Brown P. H. (2011). Climate change affects winter chill for temperate fruit and nut trees. PloS One 6, e20155. doi: 10.1371/journal.pone.0020155, PMID: 21629649 PMC3101230

[B35] Lundberg S. Lee S.-I. (2017). “A unified approach to interpreting model predictions,” in Proceedings of the 31st International Conference on Neural Information Processing Systems (Curran Associates, Long Beach, CA), 4766–4775.

[B36] Merkulov P. Latypova A. Tiurin K. Serganova M. Kirov I. (2025). DNA methylation and alternative splicing safeguard genome and transcriptome after a retrotransposition burst in arabidopsis thaliana. Int. J. Mol. Sci. 26, 4816. doi: 10.3390/ijms26104816, PMID: 40429956 PMC12112155

[B37] Nozawa K. Masuda S. Saze H. Ikeda Y. Suzuki T. Takagi H.. (2022). Epigenetic regulation of ecotype-specific expression of the heat-activated transposon ONSEN. Front. Plant Sci. 13, 899105. doi: 10.3389/fpls.2022.899105, PMID: 35923888 PMC9340270

[B38] Oberlin S. Sarazin A. Chevalier C. Voinnet O. Mari-Ordonez A. (2017). A genome-wide transcriptome and translatome analysis of arabidopsis transposons identifies a unique and conserved genome expression strategy for Ty1/Copia retroelements. Genome Res. 27, 1549–1562. doi: 10.1101/gr.220723.117, PMID: 28784835 PMC5580714

[B39] Peng Y. Yu G. I. (2024). Model multifactor analysis of soil heavy metal pollution on plant germination in Southeast Chengdu, China: Based on redundancy analysis, factor detector, and XGBoost-SHAP. Sci. Total. Environ. 954, 176605. doi: 10.1016/j.scitotenv.2024.176605, PMID: 39349201

[B40] Pietzenuk B. Markus C. Gaubert H. Bagwan N. Merotto A. Bucher E.. (2016). Recurrent evolution of heat-responsiveness in Brassicaceae COPIA elements. Genome Biol. 17, 209. doi: 10.1186/s13059-016-1072-3, PMID: 27729060 PMC5059998

[B41] Prudencio Á.S. Werner O. Martínez-García P. J. Dicenta F. Ros R. M. Martínez-Gómez P. (2018). DNA methylation analysis of dormancy release in almond (*Prunus dulcis*) flower buds using epi-genotyping by sequencing. Int. J. Mol. Sci. 19, 3542. doi: 10.3390/ijms19113542, PMID: 30423798 PMC6274898

[B42] Raihan M. J. Khan M. A.-M. Kee S.-H. Nahid A.-A. (2023). Detection of the chronic kidney disease using xgboost classifier and explaining the influence of the attributes on the model using shap. Sci. Rep. 13, 6263. doi: 10.1038/s41598-023-33525-0, PMID: 37069256 PMC10110580

[B43] Rothkegel K. Sánchez E. Montes C. Greve M. Tapia S. Bravo S.. (2017). DNA methylation and small interference RNAs participate in the regulation of MADS-box genes involved in dormancy in sweet cherry (*Prunus avium* L.). Tree Physiol. 37, 1739–1751. doi: 10.1093/treephys/tpx055, PMID: 28541567

[B44] Rothkegel K. Sandoval P. Soto E. Lissette U. Riveros A. Lillo V.. (2020). Dormant but active: chilling accumulation modulates the epigenome and transcriptome of Prunus avium during bud dormancy. Front. Plant Sci. 11. doi: 10.3389/fpls.2020.01115, PMID: 32765576 PMC7380246

[B45] Smit A. F. A. Hubley R. Green P. (2019). 2013–2015. RRepeatMasker Open-4.0. Available online at: http://www.repeatmasker.org.

[B46] Soto E. Sanchez E. Nuñez C. Montes C. Rothkegel K. Andrade P.. (2022). Small RNA differential expression analysis reveals miRNAs involved in dormancy progression in sweet cherry floral buds. Plants 11, 2396. doi: 10.3390/plants11182396, PMID: 36145795 PMC9500734

[B47] Štrumbelj E. Kononenko I. (2014). Explaining prediction models and individual predictions with feature contributions. Knowl. Inf. Syst. 41, 647–665. doi: 10.1007/s10115-013-0679-x

[B48] Weinberger J. H. (1950). Chilling requirements of peach varieties. Am. Soc. Hortic. Sci. 56, 122–128.

[B49] Xu W. Thieme M. Roulin A. C. (2024). Natural diversity of heat-induced transcription of retrotransposons in Arabidopsis thaliana. Genome Biol. Evol. 16, evae242. doi: 10.1093/gbe/evae242, PMID: 39523776 PMC11580521

[B50] Yang Q. Gao Y. Wu X. Moriguchi T. Bai S. Teng Y. (2021). Bud endodormancy in deciduous fruit trees: advances and prospects. Hortic. Res. 8, 1–11. doi: 10.1038/s41438-021-00575-2, PMID: 34078882 PMC8172858

[B51] Yoshida N. Yanai Y. Chen L. Kato Y. Hiratsuka J. Miwa T.. (2001). EMBRYONIC FLOWER2, a novel polycomb group protein homolog, mediates shoot development and flowering in Arabidopsis. Plant Cell 13, 2471–2481., PMID: 11701882 10.1105/tpc.010227 PMC139465

[B52] Zhang Z. Li Y. Xie L. Li S. Feng H. Siddique K. H.. (2024). Game analysis of future rice yield changes in China based on explainable machine-learning and planting date optimization. Field Crop Res. 317, 109557. doi: 10.1007/s13369-022-06560-8

[B53] Zhang Z. Zhuo X. Zhao K. Zheng T. Han Y. Yuan C.. (2018). Transcriptome profiles reveal the crucial roles of hormone and sugar in the bud dormancy of Prunus mume. Sci. Rep. 8, 5090. doi: 10.1038/s41598-018-23108-9, PMID: 29572446 PMC5865110

[B54] Zhou L. Ng H. K. Drautz-Moses D. I. Schuster S. C. Beck S. Kim C.. (2019). Systematic evaluation of library preparation methods and sequencing platforms for high-throughput whole genome bisulfite sequencing. Sci. Rep. 9, 1–16. doi: 10.1038/s41598-019-46875-5, PMID: 31316107 PMC6637168

[B55] Zhu H. Chen P. Y. Zhong S. Dardick C. Callahan A. An Y. Q.. (2020). Thermal-responsive genetic and epigenetic regulation of DAM cluster controlling dormancy and chilling requirement in peach floral buds. Hortic. Res. 7, 114. doi: 10.1038/s41438-020-0336-y, PMID: 32821397 PMC7395172

