# Supplementary material for: Uncovering dormancy stage predictors in sweet cherry through DNA methylation and machine learning integration
Source: Front Plant Sci. 2025 Sep 4;16:1659345. doi: 10.3389/fpls.2025.1659345 (PMC12443855; doi:10.3389/fpls.2025.1659345)
Supplement: Supplementary file 1 [file DataSheet1.docx]

Supplementary Material

# Supplementary Tables

## Table 1: Description of the bisulfite sequencing (BS-Seq) data of all paired-end libraries individually. Quality statistics of raw and after trimming datasets.

| **Sample** | **Raw Data** | | | | | **Post Trimmed** | | | | |
| --- | --- | --- | --- | --- | --- | --- | --- | --- | --- | --- |
|  | **Total Sequences** | **Total Bases (Gbp)** | **Coverage** | **Sequence length (bp)** | **%GC** | **Total Sequences** | **Total Bases (Gbp)** | **Coverage** | **Sequence length (bp)** | **%GC** |
| K_0CH_R1_1 | 40,951,722 | 5.1 | 15X | 126 | 32 | 30,573,577 | 3.7 | 11X | 100-126 | 30 |
| K_0CH_R1_2 | 40,951,722 | 5.1 | 15X | 126 | 32 | 30,573,577 | 3.7 | 11X | 100-126 | 29 |
| K_0CH_R2_1 | 41,341,370 | 5.2 | 15X | 126 | 32 | 31,241,661 | 3.8 | 11X | 100-126 | 29 |
| K_0CH_R2_2 | 41,341,370 | 5.2 | 15X | 126 | 32 | 31,241,661 | 3.8 | 11X | 100-126 | 29 |
| K_0CH_R3_1 | 34,900,265 | 4.3 | 12X | 126 | 32 | 25,816,341 | 3.1 | 9X | 100-126 | 29 |
| K_0CH_R3_2 | 34,900,265 | 4.3 | 12X | 126 | 31 | 25,816,341 | 3.1 | 9X | 100-126 | 28 |
| K_443CH_R1_1 | 28,824,580 | 3.6 | 10X | 126 | 30 | 20,326,082 | 2.4 | 7X | 100-126 | 28 |
| K_443CH_R1_2 | 28,824,580 | 3.6 | 10X | 126 | 31 | 20,326,082 | 2.4 | 7X | 100-126 | 28 |
| K_443CH_R2_1 | 31,294,993 | 3.9 | 11X | 126 | 31 | 21,878,529 | 2.6 | 8X | 100-126 | 28 |
| K_443CH_R2_2 | 31,294,993 | 3.9 | 11X | 126 | 31 | 21,878,529 | 2.6 | 8X | 100-126 | 28 |
| K_443CH_R3_1 | 32,985,376 | 4.1 | 12X | 126 | 31 | 22,137,086 | 2.6 | 8X | 100-126 | 28 |
| K_443CH_R3_2 | 32,985,376 | 4.1 | 12X | 126 | 31 | 22,137,086 | 2.7 | 8X | 100-126 | 28 |
| K_1295CH_R1_1 | 45,934,554 | 5.7 | 17X | 126 | 31 | 30,552,805 | 3.7 | 11X | 100-126 | 28 |
| K_1295CH_R1_2 | 45,934,554 | 5.7 | 17X | 126 | 31 | 30,552,805 | 3.7 | 11X | 100-126 | 28 |
| K_1295CH_R2_1 | 29,606,852 | 3.7 | 11X | 126 | 31 | 21,268,868 | 2.5 | 7X | 100-126 | 28 |
| K_1295CH_R2_2 | 29,606,852 | 3.7 | 11X | 126 | 31 | 21,268,868 | 2.6 | 8X | 100-126 | 28 |
| K_1295CH_R3_1 | 26,536,816 | 3.3 | 10X | 126 | 32 | 17,389,276 | 2.1 | 6X | 100-126 | 29 |
| K_1295CH_R3_2 | 26,536,816 | 3.3 | 10X | 126 | 32 | 17,389,276 | 2.1 | 6X | 100-126 | 29 |
| K_1637CH_R1_1 | 28,969,869 | 3.6 | 10X | 126 | 31 | 20,451,389 | 2.4 | 7X | 100-126 | 28 |
| K_1637CH_R1_2 | 28,969,869 | 3.6 | 10X | 126 | 31 | 20,451,389 | 2.4 | 7X | 100-126 | 28 |
| K_1637CH_R2_1 | 35,712,700 | 4.4 | 13X | 126 | 31 | 25,272,899 | 3.0 | 9X | 100-126 | 29 |
| K_1637CH_R2_2 | 35,712,700 | 4.4 | 13X | 126 | 31 | 25,272,899 | 3.0 | 9X | 100-126 | 28 |
| K_1637CH_R3_1 | 32,034,979 | 4.0 | 12X | 126 | 31 | 22,531,522 | 2.7 | 8X | 100-126 | 29 |
| K_1637CH_R3_2 | 32,034,979 | 4.0 | 12X | 126 | 31 | 22,531,522 | 2.7 | 8X | 100-126 | 29 |
| R_200_R1_1 | 76,316,154 | 11.4 | 33X | 150 | 22 | 34,803,377 | 5.1 | 15X | 100-150 | 22 |
| R_200_R1_2 | 76,316,154 | 11.4 | 33X | 150 | 25 | 34,803,377 | 4.8 | 14X | 100-142 | 22 |
| R_200_R2_1 | 66,816,102 | 10.0 | 29X | 150 | 22 | 30,959,918 | 4.5 | 13X | 100-150 | 22 |
| R_200_R2_2 | 66,816,102 | 10.0 | 29X | 150 | 25 | 30,959,918 | 4.3 | 12X | 100-142 | 22 |
| R_200_R3_1 | 73,555,527 | 11.0 | 32X | 150 | 22 | 28,601,960 | 4.2 | 12X | 100-150 | 22 |
| R_200_R3_2 | 73,555,527 | 11.0 | 32X | 150 | 25 | 28,601,960 | 4.0 | 12X | 100-142 | 22 |
| R_1160_R1_1 | 72,983,024 | 10.9 | 32X | 150 | 22 | 33,177,568 | 4.9 | 14X | 100-150 | 22 |
| R_1160_R1_2 | 72,983,024 | 10.9 | 32X | 150 | 25 | 33,177,568 | 4.6 | 13X | 100-142 | 22 |
| R_1160_R2_1 | 67,235,013 | 10.0 | 29X | 150 | 22 | 28,875,742 | 4.2 | 12X | 100-150 | 21 |
| R_1160_R2_2 | 67,235,013 | 10.0 | 29X | 150 | 25 | 28,875,742 | 4.0 | 12X | 100-142 | 22 |
| R_1160_R3_1 | 76,697,173 | 11.5 | 33X | 150 | 21 | 34,771,899 | 5.1 | 15X | 100-150 | 21 |
| R_1160_R3_2 | 76,697,173 | 11.5 | 33X | 150 | 25 | 34,771,899 | 4.8 | 14X | 100-142 | 21 |
| R_1700_R1_1 | 72,159,567 | 10.8 | 31X | 150 | 22 | 32,315,588 | 4.7 | 14X | 100-150 | 22 |
| R_1700_R1_2 | 72,159,567 | 10.8 | 31X | 150 | 25 | 32,315,588 | 4.5 | 13X | 100-142 | 22 |
| R_1700_R3_1 | 87,268,722 | 13.0 | 38X | 150 | 22 | 39,850,419 | 5.9 | 17X | 100-150 | 22 |
| R_1700_R3_2 | 87,268,722 | 13.0 | 38X | 150 | 25 | 39,850,419 | 5.5 | 16X | 100-142 | 22 |
| RE_202_1b_R1 | 74,897,228 | 11.2 | 33X | 150 | 23 | 63,228,900 | 9.3 | 27X | 100-150 | 23 |
| RE_202_1b_R2 | 74,897,228 | 11.2 | 33X | 150 | 26 | 63,228,900 | 8.8 | 26X | 100-142 | 23 |
| RE_202_2b_R1 | 68,776,715 | 10.3 | 30X | 150 | 23 | 58,078,780 | 8.5 | 25X | 100-150 | 23 |
| RE_202_2b_R2 | 68,776,715 | 10.3 | 30X | 150 | 26 | 58,078,780 | 8.1 | 24X | 100-142 | 23 |
| RE_202_3b_R1 | 72,173,567 | 10.8 | 31X | 150 | 23 | 61,637,280 | 9.1 | 26X | 100-150 | 23 |
| RE_202_3b_R2 | 72,173,567 | 10.8 | 31X | 150 | 26 | 61,637,280 | 8.6 | 25X | 100-142 | 23 |
| RE_463_1b_R1 | 81,871,088 | 12.2 | 35X | 150 | 22 | 66,906,086 | 9.9 | 29X | 100-150 | 22 |
| RE_463_1b_R2 | 81,871,088 | 12.2 | 35X | 150 | 25 | 66,906,086 | 9.3 | 27X | 100-142 | 22 |
| RE_463_2b_R1 | 105,380,440 | 15.8 | 46X | 150 | 22 | 86,233,773 | 12.7 | 37X | 100-150 | 22 |
| RE_463_2b_R2 | 105,380,440 | 15.8 | 46X | 150 | 25 | 86,233,773 | 12.0 | 35X | 100-142 | 22 |
| RE_463_3b_R1 | 72,366,983 | 10.8 | 31X | 150 | 22 | 59,677,126 | 8.8 | 26X | 100-150 | 22 |
| RE_463_3b_R2 | 72,366,983 | 10.8 | 31X | 150 | 25 | 59,677,126 | 8.3 | 24X | 100-142 | 22 |
| RE_769_2b_R1 | 69,209,289 | 10.3 | 30X | 150 | 23 | 58,511,144 | 8.6 | 25X | 100-150 | 23 |
| RE_769_2b_R2 | 69,209,289 | 10.3 | 30X | 150 | 26 | 58,511,144 | 8.1 | 24X | 100-142 | 23 |
| RE_769_3b_R1 | 78,382,534 | 11.7 | 34X | 150 | 22 | 67,303,788 | 9.9 | 29X | 100-150 | 23 |
| RE_769_3b_R2 | 78,382,534 | 11.7 | 34X | 150 | 26 | 67,303,788 | 9.4 | 27X | 100-142 | 23 |
| RE_769_4b_R1 | 71,198,887 | 10.6 | 31X | 150 | 22 | 61,591,376 | 9.1 | 26X | 100-150 | 23 |
| RE_769_4b_R2 | 71,198,887 | 10.6 | 31X | 150 | 26 | 61,591,376 | 8.6 | 25X | 100-142 | 23 |
| RE_837_2b_R1 | 118,776,185 | 17.8 | 52X | 150 | 22 | 100,223,165 | 14.8 | 43X | 100-150 | 23 |
| RE_837_2b_R2 | 118,776,185 | 17.8 | 52X | 150 | 26 | 100,223,165 | 14.0 | 41X | 100-142 | 23 |
| RE_837_3b_R1 | 74,975,504 | 11.2 | 33X | 150 | 22 | 64,384,919 | 9.5 | 28X | 100-150 | 22 |
| RE_837_3b_R2 | 74,975,504 | 11.2 | 33X | 150 | 25 | 64,384,919 | 9.0 | 26X | 100-142 | 23 |
| RE_837_4b_R1 | 199,690,298 | 29.9 | 87X | 150 | 22 | 172,240,166 | 25.5 | 74X | 100-150 | 22 |
| RE_837_4b_R2 | 199,690,298 | 29.9 | 87X | 150 | 25 | 172,240,166 | 24.1 | 70X | 100-142 | 23 |
| RE_959_1b_R1 | 73,137,278 | 10.9 | 32X | 150 | 23 | 62,651,035 | 9.2 | 27X | 100-150 | 23 |
| RE_959_1b_R2 | 73,137,278 | 10.9 | 32X | 150 | 26 | 62,651,035 | 8.7 | 25X | 100-142 | 23 |
| RE_959_2b_R1 | 69,366,663 | 10.4 | 30X | 150 | 22 | 57,720,644 | 8.5 | 25X | 100-150 | 22 |
| RE_959_2b_R2 | 69,366,663 | 10.4 | 30X | 150 | 26 | 57,720,644 | 8.0 | 23X | 100-142 | 23 |
| RE_959_3b_R1 | 69,813,855 | 10.4 | 30X | 150 | 22 | 59,450,580 | 8.8 | 26X | 100-150 | 22 |
| RE_959_3b_R2 | 69,813,855 | 10.4 | 30X | 150 | 25 | 59,450,580 | 8.3 | 24X | 100-142 | 22 |
| RM_268_1b_R1 | 69,051,817 | 10.3 | 30X | 150 | 23 | 57,727,449 | 8.5 | 25X | 100-150 | 23 |
| RM_268_1b_R2 | 69,051,817 | 10.3 | 30X | 150 | 26 | 57,727,449 | 7.7 | 22X | 100-137 | 23 |
| RM_268_2b_R1 | 78,582,496 | 11.7 | 34X | 150 | 23 | 66,463,138 | 9.8 | 28X | 100-150 | 23 |
| RM_268_2b_R2 | 78,582,496 | 11.7 | 34X | 150 | 26 | 66,463,138 | 8.9 | 26X | 100-137 | 23 |
| RM_268_4b_R1 | 67,556,536 | 10.1 | 29X | 150 | 23 | 57,418,859 | 8.5 | 25X | 100-150 | 23 |
| RM_268_4b_R2 | 67,556,536 | 10.1 | 29X | 150 | 26 | 57,418,859 | 7.7 | 22X | 100-137 | 23 |
| RM_599_1b_R1 | 72,468,837 | 10.8 | 31X | 150 | 22 | 61,424,571 | 9.1 | 26X | 100-150 | 22 |
| RM_599_1b_R2 | 72,468,837 | 10.8 | 31X | 150 | 25 | 61,424,571 | 8.3 | 24X | 100-137 | 23 |
| RM_599_3b_R1 | 70,814,825 | 10.6 | 31X | 150 | 22 | 60,183,564 | 8.9 | 26X | 100-150 | 23 |
| RM_599_3b_R2 | 70,814,825 | 10.6 | 31X | 150 | 26 | 60,183,564 | 8.1 | 24X | 100-137 | 23 |
| RM_599_4b_R1 | 71,570,564 | 10.7 | 31X | 150 | 22 | 60,177,170 | 8.9 | 26X | 100-150 | 22 |
| RM_599_4b_R2 | 71,570,564 | 10.7 | 31X | 150 | 26 | 60,177,170 | 8.1 | 24X | 100-137 | 23 |
| RM_973_1b_R1 | 79,391,251 | 11.9 | 35X | 150 | 22 | 65,411,870 | 9.7 | 28X | 100-150 | 22 |
| RM_973_1b_R2 | 79,391,251 | 11.9 | 35X | 150 | 25 | 65,411,870 | 8.8 | 26X | 100-137 | 22 |
| RM_973_2b_R1 | 70,680,205 | 10.6 | 31X | 150 | 22 | 59,490,350 | 8.8 | 26X | 100-150 | 22 |
| RM_973_2b_R2 | 70,680,205 | 10.6 | 31X | 150 | 25 | 59,490,350 | 8.0 | 23X | 100-137 | 23 |
| RM_973_3b_R1 | 104,477,421 | 15.6 | 45X | 150 | 23 | 88,358,235 | 13.1 | 38X | 100-150 | 23 |
| RM_973_3b_R2 | 104,477,421 | 15.6 | 45X | 150 | 26 | 88,358,235 | 11.9 | 35X | 100-137 | 23 |
| RM_1234_1b_R1 | 101,372,138 | 15.2 | 44X | 150 | 22 | 85,702,768 | 12.7 | 37X | 100-150 | 22 |
| RM_1234_1b_R2 | 101,372,138 | 15.2 | 44X | 150 | 25 | 85,702,768 | 11.5 | 33X | 100-137 | 23 |
| RM_1234_3b_R1 | 71,066,593 | 10.6 | 31X | 150 | 22 | 57,987,441 | 8.5 | 25X | 100-150 | 22 |
| RM_1234_3b_R2 | 71,066,593 | 10.6 | 31X | 150 | 25 | 57,987,441 | 7.8 | 23X | 100-137 | 22 |
| RM_1234_4b_R1 | 70,908,716 | 10.6 | 31X | 150 | 22 | 60,204,212 | 8.9 | 26X | 100-150 | 22 |
| RM_1234_4b_R2 | 70,908,716 | 10.6 | 31X | 150 | 25 | 60,204,212 | 8.1 | 24X | 100-137 | 23 |
| SM_268_2b_R1 | 70,786,049 | 10.6 | 31X | 150 | 23 | 70,757,227 | 10.5 | 30X | 107-150 | 23 |
| SM_268_2b_R2 | 70,786,049 | 10.6 | 31X | 150 | 27 | 70,757,227 | 10.6 | 31X | 131-150 | 27 |
| SM_268_3b_R1 | 88,567,795 | 13.2 | 38X | 150 | 22 | 88,567,781 | 13.2 | 38X | 109-150 | 22 |
| SM_268_3b_R2 | 88,567,795 | 13.2 | 38X | 150 | 25 | 88,567,781 | 13.2 | 38X | 131-150 | 25 |
| SM_268_4b_R1 | 69,409,140 | 10.4 | 30X | 150 | 23 | 69,402,020 | 10.3 | 30X | 101-150 | 23 |
| SM_268_4b_R2 | 69,409,140 | 10.4 | 30X | 150 | 26 | 69,402,020 | 10.4 | 30X | 131-150 | 26 |
| SM_599_1b_R1 | 70,926,665 | 10.6 | 31X | 150 | 23 | 70,926,634 | 10.6 | 31X | 108-150 | 23 |
| SM_599_1b_R2 | 70,926,665 | 10.6 | 31X | 150 | 26 | 70,926,634 | 10.6 | 31X | 117-150 | 26 |
| SM_599_2b_R1 | 71,629,679 | 10.7 | 31X | 150 | 23 | 71,629,679 | 10.7 | 31X | 109-150 | 23 |
| SM_599_2b_R2 | 71,629,679 | 10.7 | 31X | 150 | 26 | 71,629,679 | 10.7 | 31X | 131-150 | 26 |
| SM_599_3b_R1 | 69,816,779 | 10.4 | 30X | 150 | 23 | 69,795,131 | 10.4 | 30X | 107-150 | 23 |
| SM_599_3b_R2 | 69,816,779 | 10.4 | 30X | 150 | 26 | 69,795,131 | 10.4 | 30X | 131-150 | 26 |
| SM_973_1b_R1 | 69,975,922 | 10.4 | 30X | 150 | 23 | 69,924,255 | 10.4 | 30X | 100-150 | 23 |
| SM_973_1b_R2 | 69,975,922 | 10.4 | 30X | 150 | 26 | 69,924,255 | 10.4 | 30X | 131-150 | 26 |
| SM_973_2b_R1 | 69,703,027 | 10.4 | 30X | 150 | 22 | 69,703,026 | 10.4 | 30X | 109-150 | 22 |
| SM_973_2b_R2 | 69,703,027 | 10.4 | 30X | 150 | 26 | 69,703,026 | 10.4 | 30X | 132-150 | 26 |
| SM_973_4b_R1 | 75,480,853 | 11.3 | 33X | 150 | 23 | 75,480,810 | 11.2 | 33X | 109-150 | 23 |
| SM_973_4b_R2 | 75,480,853 | 11.3 | 33X | 150 | 26 | 75,480,810 | 11.3 | 33X | 120-150 | 26 |
| SM_1234_1b_R1 | 82,633,970 | 12.3 | 36X | 150 | 22 | 82,589,842 | 12.3 | 36X | 106-150 | 22 |
| SM_1234_1b_R2 | 82,633,970 | 12.3 | 36X | 150 | 26 | 82,589,842 | 12.3 | 36X | 128-150 | 26 |
| SM_1234_3b_R1 | 68,074,609 | 10.2 | 30X | 150 | 22 | 68,060,623 | 10.1 | 29X | 100-150 | 22 |
| SM_1234_3b_R2 | 68,074,609 | 10.2 | 30X | 150 | 26 | 68,060,623 | 10.2 | 30X | 131-150 | 26 |
| SM_1234_4b_R1 | 73,712,628 | 11.0 | 32X | 150 | 22 | 73,681,850 | 11.0 | 32X | 107-150 | 22 |
| SM_1234_4b_R2 | 73,712,628 | 11.0 | 32X | 150 | 26 | 73,681,850 | 11.0 | 32X | 122-150 | 26 |
| RD_0CH_R1_1 | 38,702,909 | 4.8 | 14X | 126 | 32 | 27,451,414 | 3.3 | 10X | 100-126 | 29 |
| RD_0CH_R1_2 | 38,702,909 | 4.8 | 14X | 126 | 32 | 27,451,414 | 3.3 | 10X | 100-126 | 29 |
| RD_0CH_R2_1 | 29,608,334 | 3.7 | 11X | 126 | 32 | 20,603,656 | 2.5 | 7X | 100-126 | 29 |
| RD_0CH_R2_2 | 29,608,334 | 3.7 | 11X | 126 | 32 | 20,603,656 | 2.5 | 7X | 100-126 | 29 |
| RD_0CH_R3_1 | 39,308,752 | 4.9 | 14X | 126 | 33 | 28,397,693 | 3.4 | 10X | 100-126 | 30 |
| RD_0CH_R3_2 | 39,308,752 | 4.9 | 14X | 126 | 33 | 28,397,693 | 3.4 | 10X | 100-126 | 30 |
| RD_173CH_R1_1 | 30,348,974 | 3.8 | 11X | 126 | 31 | 21,258,750 | 2.5 | 7X | 100-126 | 28 |
| RD_173CH_R1_2 | 30,348,974 | 3.8 | 11X | 126 | 31 | 21,258,750 | 2.5 | 7X | 100-126 | 28 |
| RD_173CH_R2_1 | 26,040,791 | 3.2 | 9X | 126 | 31 | 18,450,233 | 2.2 | 6X | 100-126 | 28 |
| RD_173CH_R2_2 | 26,040,791 | 3.2 | 9X | 126 | 31 | 18,450,233 | 2.2 | 6X | 100-126 | 28 |
| RD_173CH_R3_1 | 37,471,126 | 4.7 | 14X | 126 | 31 | 27,098,918 | 3.3 | 10X | 100-126 | 29 |
| RD_173CH_R3_2 | 37,471,126 | 4.7 | 14X | 126 | 31 | 27,098,918 | 3.3 | 10X | 100-126 | 28 |
| RD_348CH_R1_1 | 35,609,904 | 4.4 | 13X | 126 | 31 | 25,251,890 | 3.0 | 9X | 100-126 | 28 |
| RD_348CH_R1_2 | 35,609,904 | 4.4 | 13X | 126 | 31 | 25,251,890 | 3.0 | 9X | 100-126 | 28 |
| RD_348CH_R2_1 | 29,306,540 | 3.6 | 10X | 126 | 31 | 20,741,826 | 2.5 | 7X | 100-126 | 29 |
| RD_348CH_R2_2 | 29,306,540 | 3.6 | 10X | 126 | 31 | 20,741,826 | 2.5 | 7X | 100-126 | 29 |
| RD_348CH_R3_1 | 30,252,286 | 3.8 | 11X | 126 | 32 | 19,925,374 | 2.4 | 7X | 100-126 | 29 |
| RD_348CH_R3_2 | 30,252,286 | 3.8 | 11X | 126 | 32 | 19,925,374 | 2.4 | 7X | 100-126 | 29 |
| RD_516CH_R1_1 | 30,538,061 | 3.8 | 11X | 126 | 31 | 21,353,759 | 2.6 | 8X | 100-126 | 28 |
| RD_516CH_R1_2 | 30,538,061 | 3.8 | 11X | 126 | 31 | 21,353,759 | 2.6 | 8X | 100-126 | 28 |
| RD_516CH_R2_1 | 26,452,246 | 3.3 | 10X | 126 | 31 | 18,148,481 | 2.2 | 6X | 100-126 | 29 |
| RD_516CH_R2_2 | 26,452,246 | 3.3 | 10X | 126 | 31 | 18,148,481 | 2.2 | 6X | 100-126 | 28 |
| RD_516CH_R3_1 | 28,431,439 | 3.5 | 10X | 126 | 31 | 18,864,207 | 2.2 | 6X | 100-126 | 28 |
| RD_516CH_R3_2 | 28,431,439 | 3.5 | 10X | 126 | 31 | 18,864,207 | 2.2 | 6X | 100-126 | 28 |

## Table 2. Parameters of whole genome bisulfite sequencing (WGBS) libraries mapped against reference *P. avium* genome Tieton v2.0 for each chilling hour condition and three biological replicates (R).

| **Sample** | **Mapping efficiency** | **Sequence pairs analyzed** | **Uniquely mapped reads** | **Multiple mapped reads** | **Reads with no alignment** | **Genome (344 Mbp) coverage of uniquely mapped reads** |
| --- | --- | --- | --- | --- | --- | --- |
| K_0_R1 | 56.10% | 30,573,577 | 17,162,719 | 9,547,792 | 3,863,066 | 7X |
| K_0_R2 | 55.50% | 31,241,661 | 17,326,558 | 9,652,877 | 4,262,226 | 7X |
| K_0_R3 | 56.50% | 25,816,341 | 14,591,560 | 7,694,401 | 3,530,380 | 6X |
| K_443_R1 | 48.90% | 20,326,082 | 9,940,233 | 5,165,223 | 5,220,626 | 4X |
| K_443_R2 | 47.90% | 21,878,529 | 10,490,360 | 5,488,382 | 5,899,787 | 5X |
| K_443_R3 | 48.20% | 22,137,086 | 10,668,944 | 5,556,875 | 5,911,267 | 5X |
| K_1295_R1 | 48.30% | 30,552,805 | 14,760,271 | 7,794,590 | 7,997,944 | 6X |
| K_1295_R2 | 48.10% | 21,268,868 | 10,226,169 | 5,396,954 | 5,645,745 | 4X |
| K_1295_R3 | 47.60% | 17,389,276 | 8,272,881 | 4,707,915 | 4,408,480 | 4X |
| K_1637_R1 | 49.10% | 20,451,389 | 10,050,363 | 5,121,340 | 5,279,686 | 4X |
| K_1637_R2 | 49.00% | 25,272,899 | 12,383,830 | 6,430,677 | 6,458,392 | 5X |
| K_1637_R3 | 47.80% | 22,531,522 | 10,763,342 | 5,973,215 | 5,794,965 | 5X |
| R_200_R1 | 65.60% | 34,803,377 | 22,821,926 | 6,215,211 | 5,766,240 | 10X |
| R_200_R2 | 65.40% | 30,959,918 | 20,233,694 | 5,906,567 | 4,819,657 | 9X |
| R_200_R3 | 68.90% | 28,601,960 | 19,716,725 | 5,269,173 | 3,616,062 | 9X |
| R_1160_R1 | 66.10% | 33,177,568 | 21,915,247 | 6,006,992 | 5,255,329 | 9X |
| R_1160_R2 | 68.10% | 28,875,742 | 19,677,234 | 5,223,858 | 3,974,650 | 9X |
| R_1160_R3 | 68.30% | 34,771,899 | 23,753,410 | 6,194,741 | 4,823,748 | 10X |
| R_1700_R1 | 66.60% | 32,315,588 | 21,514,673 | 5,568,464 | 5,232,451 | 9X |
| R_1700_R3 | 66.90% | 39,850,419 | 26,674,912 | 7,843,273 | 5,332,234 | 12X |
| RE_202_R1 | 62.90% | 63,228,900 | 63,228,900 | 12,205,100 | 11,231,795 | 27X |
| RE_202_R2 | 63.20% | 58,078,780 | 36,685,612 | 11,358,528 | 10,034,640 | 16X |
| RE_202_R3 | 63.80% | 61,637,280 | 39,344,009 | 11,284,108 | 11,009,163 | 17X |
| RE_463_R1 | 67.80% | 66,906,086 | 45,377,215 | 11,649,704 | 9,879,167 | 20X |
| RE_463_R2 | 68.00% | 86,233,773 | 58,602,146 | 15,221,578 | 12,410,049 | 25X |
| RE_463_R3 | 66.10% | 59,677,126 | 39,472,025 | 10,131,994 | 10,073,107 | 17X |
| RE_769_R1 | 64.40% | 58,511,144 | 37,660,345 | 10,210,292 | 10,640,507 | 16X |
| RE_769_R2 | 65.90% | 67,303,788 | 44,383,285 | 12,010,371 | 10,910,132 | 19X |
| RE_769_R3 | 65.90% | 61,591,376 | 40,592,978 | 11,116,347 | 9,882,051 | 18X |
| RE_837_R1 | 63.30% | 100,223,165 | 63,400,190 | 18,023,421 | 18,799,554 | 27X |
| RE_837_R2 | 66.00% | 64,384,919 | 42,483,210 | 11,320,630 | 10,581,079 | 18X |
| RE_837_R3 | 66.50% | 172,240,166 | 114,569,140 | 30,507,270 | 27,163,756 | 50X |
| RE_959_R1 | 61.40% | 62,651,035 | 38,440,848 | 11,776,844 | 12,433,343 | 17X |
| RE_959_R2 | 63.60% | 57,720,644 | 36,696,228 | 10,613,573 | 10,410,843 | 16X |
| RE_959_R3 | 63.00% | 59,450,580 | 37,458,973 | 11,014,817 | 10,976,790 | 16X |
| RM_268_R1 | 48.20% | 68,924,039 | 33,191,052 | 7,730,869 | 28,002,118 | 14X |
| RM_268_R2 | 48.90% | 78,447,892 | 38,335,089 | 8,881,333 | 31,231,470 | 17X |
| RM_268_R3 | 48.90% | 67,443,320 | 32,971,830 | 7,980,116 | 26,491,374 | 14X |
| RM_599_R1 | 47.60% | 72,336,876 | 34,444,037 | 7,708,316 | 30,184,523 | 15X |
| RM_599_R2 | 47.60% | 70,686,907 | 33,618,360 | 8,218,316 | 28,850,231 | 15X |
| RM_599_R3 | 47.60% | 71,418,951 | 33,959,805 | 8,040,674 | 29,418,472 | 15X |
| RM_973_R1 | 47.30% | 79,250,134 | 37,453,554 | 7,991,012 | 33,805,568 | 16X |
| RM_973_R2 | 47.20% | 70,547,704 | 33,328,827 | 7,604,169 | 29,614,708 | 14X |
| RM_973_R3 | 47.30% | 104,291,854 | 49,340,567 | 11,596,089 | 43,355,198 | 21X |
| RM_1234_R1 | 47.00% | 101,175,380 | 47,536,314 | 11,115,616 | 42,523,450 | 21X |
| RM_1234_R2 | 44.40% | 70,928,879 | 31,489,600 | 7,267,650 | 32,171,629 | 14X |
| RM_1234_R3 | 46.50% | 70,782,801 | 32,917,222 | 8,063,862 | 29,801,717 | 14X |
| SM_268_R1 | 56.00% | 53,489,839 | 29,935,614 | 7,591,444 | 15,962,781 | 13X |
| SM_268_R2 | 53.70% | 65,906,366 | 35,394,950 | 7,825,275 | 22,686,141 | 15X |
| SM_268_R3 | 55.50% | 52,558,862 | 29,149,027 | 6,618,835 | 16,791,000 | 13X |
| SM_599_R1 | 54.00% | 52,902,629 | 28,573,858 | 6,947,142 | 17,381,629 | 12X |
| SM_599_R2 | 54.60% | 53,622,473 | 29,286,256 | 7,485,813 | 16,850,404 | 13X |
| SM_599_R3 | 53.80% | 52,614,945 | 28,297,257 | 7,216,679 | 17,101,009 | 12X |
| SM_973_R1 | 53.00% | 51,925,074 | 27,524,881 | 7,056,318 | 17,343,875 | 12X |
| SM_973_R2 | 55.10% | 52,429,265 | 28,864,510 | 6,752,369 | 16,812,386 | 12X |
| SM_973_R3 | 53.80% | 55,306,847 | 29,747,093 | 6,908,047 | 18,651,707 | 13X |
| SM_1234_R1 | 54.90% | 60,628,889 | 33,273,746 | 7,813,595 | 19,541,548 | 14X |
| SM_1234_R2 | 53.10% | 51,015,798 | 27,095,000 | 6,779,294 | 17,141,504 | 12X |
| SM_1234_R3 | 53.20% | 53,822,904 | 28,609,294 | 7,083,246 | 18,130,364 | 12X |
| RD_0_R1 | 55.30% | 27,451,414 | 15,168,007 | 8,434,935 | 3,848,472 | 7X |
| RD_0_R2 | 55.20% | 20,603,656 | 11,368,352 | 6,622,958 | 2,612,346 | 5X |
| RD_0_R3 | 55.40% | 28,397,693 | 15,738,180 | 9,235,454 | 3,424,059 | 7X |
| RD_173_R1 | 48.00% | 21,258,750 | 10,210,471 | 5,386,052 | 5,662,227 | 4X |
| RD_173_R2 | 48.30% | 18,450,233 | 8,915,643 | 4,724,469 | 4,810,121 | 4X |
| RD_173_R3 | 47.70% | 27,098,918 | 12,926,565 | 7,066,966 | 7,105,387 | 6X |
| RD_348_R1 | 48.00% | 25,251,890 | 12,122,508 | 6,483,036 | 6,646,346 | 5X |
| RD_348_R2 | 47.50% | 20,741,826 | 9,860,633 | 5,505,961 | 5,375,232 | 4X |
| RD_348_R3 | 46.50% | 19,925,374 | 9,272,698 | 5,339,630 | 5,313,046 | 4X |
| RD_516_R1 | 47.30% | 21,353,759 | 10,103,716 | 5,498,658 | 5,751,385 | 4X |
| RD_516_R2 | 46.90% | 18,148,481 | 8,518,652 | 4,715,703 | 4,914,126 | 4X |
| RD_516_R3 | 47.30% | 18,864,207 | 8,918,198 | 4,915,513 | 5,030,496 | 4X |

## Table 3. Absolute methylation levels for each library at a corresponding chilling accumulation (in chilling hours) and replicate. mCG, mCHG and mCHH (H= C, T or A) represent the three cytosine methylation contexts as a percentage of methylation*. Color scale indicates: higher (red), medium (white), or lower (blue) methylation values in the corresponding cytosine context.

| **Sample** | **Total C's analyzed** | **%mCG** | **%mCHG** | **%mCHH** |
| --- | --- | --- | --- | --- |
| K_0_R1 | 828,059,223 | 83.3 | 67.6 | 10.9 |
| K_0_R2 | 837,250,421 | 82.3 | 66.4 | 10.5 |
| K_0_R3 | 686,959,550 | 76.4 | 59.6 | 9.7 |
| K_443_R1 | 474,996,578 | 79.0 | 61.6 | 9.9 |
| K_443_R2 | 501,471,983 | 79.1 | 62.0 | 10.3 |
| K_443_R3 | 511,314,899 | 79.9 | 62.6 | 10.4 |
| K_1295_R1 | 702,153,809 | 78.5 | 61.5 | 10.5 |
| K_1295_R2 | 490,362,323 | 80.0 | 63.0 | 10.9 |
| K_1295_R3 | 403,143,228 | 83.9 | 68.4 | 11.2 |
| K_1637_R1 | 478,574,466 | 77.8 | 60.8 | 10.8 |
| K_1637_R2 | 591,177,031 | 79.3 | 62.6 | 10.6 |
| K_1637_R3 | 521,640,570 | 82.9 | 66.6 | 11.0 |
| R_200_R1 | 1,222,414,379 | 57.7 | 29.6 | 4.4 |
| R_200_R2 | 1,088,333,404 | 58.8 | 30.5 | 4.3 |
| R_200_R3 | 1,032,866,574 | 63.6 | 35.0 | 6.1 |
| R_1160_R1 | 1,164,276,049 | 60.8 | 32.8 | 4.6 |
| R_1160_R2 | 1,020,038,182 | 64.1 | 36.0 | 5.6 |
| R_1160_R3 | 1,193,905,428 | 66.7 | 39.9 | 6.4 |
| R_1700_R1 | 1,148,697,829 | 59.0 | 30.6 | 4.7 |
| R_1700_R3 | 1,400,430,319 | 67.5 | 39.0 | 6.2 |
| RE_202_R1 | 2,170,892,030 | 66.3 | 35.7 | 4.6 |
| RE_202_R2 | 2,005,128,867 | 65.6 | 35.1 | 4.3 |
| RE_202_R3 | 2,151,686,319 | 64.4 | 34.6 | 4.9 |
| RE_463_R2 | 2,041,741,607 | 62.4 | 33.3 | 4.8 |
| RE_463_R3 | 2,389,050,161 | 59.0 | 30.5 | 4.9 |
| RE_463_R4 | 2,195,250,167 | 58.7 | 30.4 | 4.9 |
| RE_769_R2 | 3,458,569,934 | 62.8 | 33.3 | 4.8 |
| RE_769_R3 | 2,304,468,247 | 58.9 | 30.3 | 4.9 |
| RE_769_R4 | 6,217,914,137 | 57.8 | 29.3 | 4.8 |
| RE_837_R1 | 2,108,876,388 | 63.1 | 33.3 | 4.3 |
| RE_837_R2 | 1,986,033,520 | 62.9 | 32.9 | 4.3 |
| RE_837_R3 | 2,032,350,760 | 62.4 | 32.2 | 3.9 |
| RE_959_R1 | 2,441,726,744 | 56.1 | 28.4 | 4.6 |
| RE_959_R2 | 3,146,263,333 | 56.4 | 28.5 | 4.6 |
| RE_959_R3 | 2,136,234,230 | 55.1 | 27.5 | 4.6 |
| RM_268_R1 | 1,882,444,694 | 65.5 | 36.2 | 7.4 |
| RM_268_R2 | 2,172,783,184 | 65.7 | 36.6 | 7.0 |
| RM_268_R4 | 1,875,860,127 | 65.4 | 36.9 | 7.1 |
| RM_599_R1 | 1,952,205,812 | 61.9 | 33.3 | 7.6 |
| RM_599_R3 | 1,911,162,419 | 64.4 | 34.8 | 7.2 |
| RM_599_R4 | 1,937,777,854 | 62.2 | 33.3 | 7.3 |
| RM_973_R1 | 2,125,544,241 | 61.8 | 33.7 | 7.1 |
| RM_973_R2 | 1,893,237,432 | 63.6 | 34.2 | 7.4 |
| RM_973_R3 | 2,811,347,645 | 65.0 | 35.7 | 7.9 |
| RM_1234_R1 | 2,697,550,784 | 63.4 | 34.4 | 6.8 |
| RM_1234_R3 | 1,792,677,779 | 62.3 | 32.7 | 6.4 |
| RM_1234_R4 | 1,872,993,011 | 64.8 | 34.9 | 6.6 |
| SM_268_R2 | 1,703,991,239 | 67.7 | 39.4 | 7.7 |
| SM_268_R3 | 2,000,999,659 | 63.5 | 35.0 | 6.8 |
| SM_268_R4 | 1,649,204,193 | 64.7 | 36.3 | 7.6 |
| SM_599_R1 | 1,620,966,259 | 64.8 | 36.4 | 7.4 |
| SM_599_R2 | 1,668,780,842 | 66.6 | 38.1 | 7.3 |
| SM_599_R3 | 1,612,220,709 | 66.1 | 37.6 | 7.4 |
| SM_973_R1 | 1,568,030,679 | 66.8 | 38.1 | 7.3 |
| SM_973_R2 | 1,637,400,627 | 63.5 | 35.3 | 7.2 |
| SM_973_R4 | 1,688,268,892 | 64.2 | 36.0 | 7.7 |
| SM_1234_R1 | 1,877,471,949 | 63.9 | 34.8 | 6.0 |
| SM_1234_R3 | 1,537,947,240 | 66.3 | 36.9 | 6.1 |
| SM_1234_R4 | 1,631,545,319 | 64.9 | 35.7 | 6.2 |
| RD_0_R1 | 718,988,657 | 77.8 | 60.9 | 9.8 |
| RD_0_R2 | 548,808,872 | 81.1 | 64.4 | 9.8 |
| RD_0_R3 | 764,384,987 | 82.6 | 66.4 | 11.2 |
| RD_173_R1 | 486,447,054 | 76.8 | 58.9 | 10.4 |
| RD_173_R2 | 427,816,213 | 78.7 | 61.2 | 10.9 |
| RD_173_R3 | 624,475,267 | 80.0 | 63.1 | 10.2 |
| RD_348_R1 | 581,885,204 | 78.5 | 61.6 | 10.4 |
| RD_348_R2 | 478,155,962 | 81.7 | 65.7 | 10.1 |
| RD_348_R3 | 450,972,369 | 82.9 | 67.3 | 11.4 |
| RD_516_R1 | 485,409,197 | 78.3 | 61.0 | 10.2 |
| RD_516_R2 | 410,171,663 | 78.9 | 61.9 | 10.2 |
| RD_516_R3 | 427,951,318 | 78.5 | 60.5 | 10.9 |

## Table 4. Distribution of transposable elements (TEs) across genomic features, grouped by TE class. Summary of the number of features (cytosine or region) overlapping transposable elements (TEs) by TE class and their genomic context. TE classes (e.g., LTR/Copia, DNA/MULE-MuDR), while columns indicate genomic features where these TEs are located: Promoter (2 kb upstream of TSS), Gene (exonic and intronic regions), Downstream (2 kb downstream 3'), and Intergenic (regions not overlapping annotated gene-proximal features). Counts represent the number of TEs found in each TE class–feature combination. Only TE classes with at least one overlapping cytosine are shown.

| **TE_Class** | **Promoter** | **Gene** | **Downstream** | **Intergenic** |
| --- | --- | --- | --- | --- |
| Unknown | 94 | 67 | 40 | 0 |
| LTR/Ty3-retrotransposons | 60 | 51 | 33 | 0 |
| LTR/Copia | 37 | 35 | 26 | 0 |
| LTR/Unknown | 19 | 11 | 19 | 0 |
| DNA/MULE-MuDR | 12 | 13 | 5 | 0 |
| LINE/L1 | 4 | 4 | 9 | 0 |
| DNA/CMC-EnSpm | 3 | 4 | 5 | 0 |
| rRNA | 6 | 0 | 3 | 0 |
| RC/Helitron | 5 | 1 | 0 | 0 |
| DNA/PIF-Harbinger | 5 | 0 | 0 | 0 |
| LTR/Caulimovirus | 2 | 1 | 1 | 0 |
| PLE/Naiad | 1 | 2 | 0 | 0 |
| SINE/tRNA | 1 | 1 | 0 | 0 |
| Simple_repeat | 1 | 1 | 0 | 0 |
| DNA/hAT-Ac | 0 | 1 | 0 | 0 |
| DNA/hAT-Blackjack | 1 | 0 | 0 | 0 |
| DNA/hAT-Tag1 | 0 | 0 | 1 | 0 |
| DNA/hAT-Tip100 | 1 | 0 | 0 | 0 |
| LTR/ERV1 | 0 | 0 | 1 | 0 |
| SINE/5S | 1 | 0 | 0 | 0 |

#
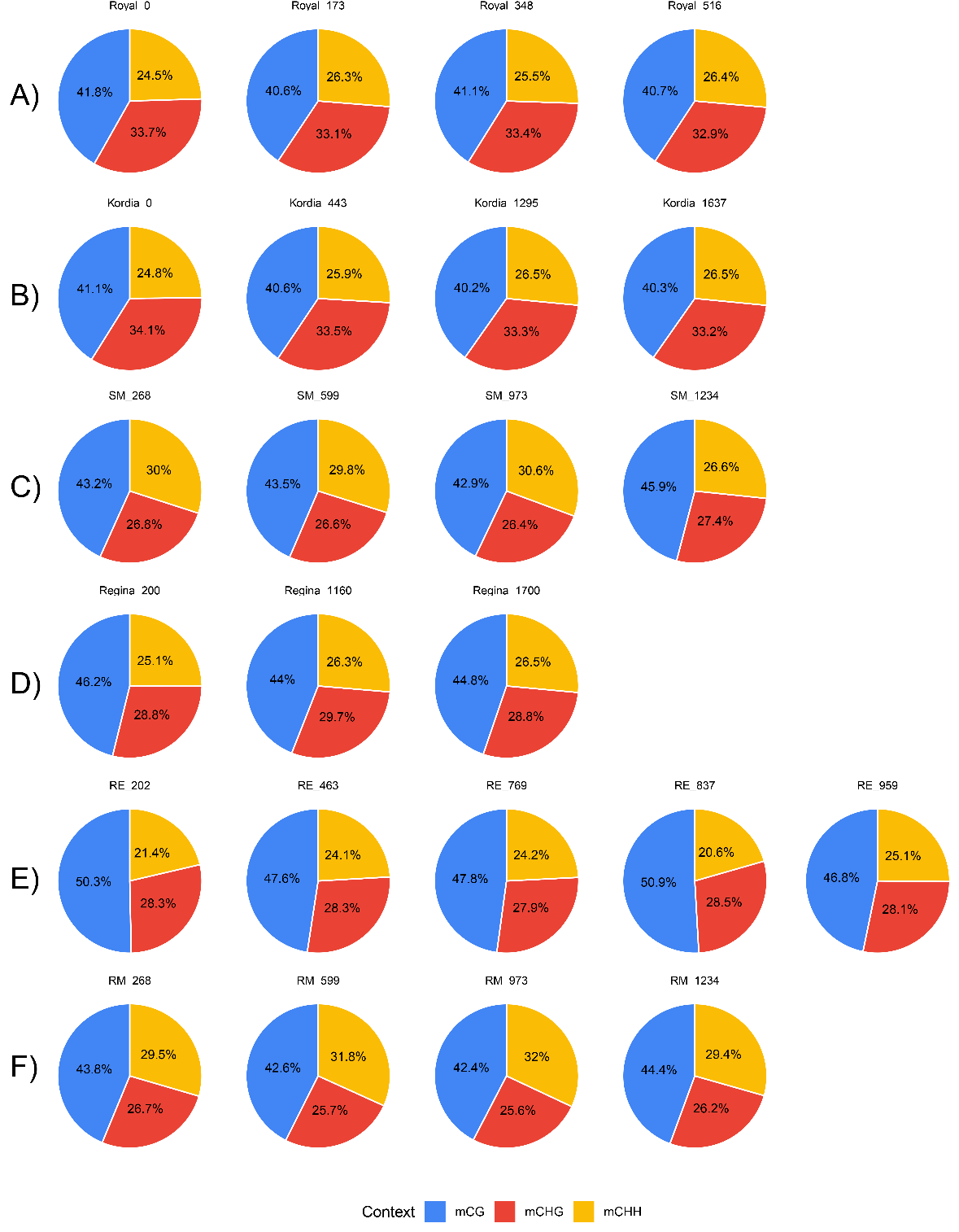
Supplementary Figures

## **Figure 1. Genome-wide methylated cytosine contexts across cultivars and experiments. Pie charts** show the relative contribution of each methylation context; mCG (blue), mCHG (red), and mCHH (yellow), to the total number of methylated cytosines per sample. Samples are grouped by cultivar and year of collection: (A) Royal Dawn (Mostazal, 2015), (B) Kordia (Quillota, 2015), (C) Santina (Morza, 2022), (D) Regina (Los Tilos, 2021), (E) Regina (Entre Ríos, 2022), and (F) Regina (Morza, 2022). Cultivars were arranged from left to right according to their increase in chill accumulation measured in chilling hours (CH).

##
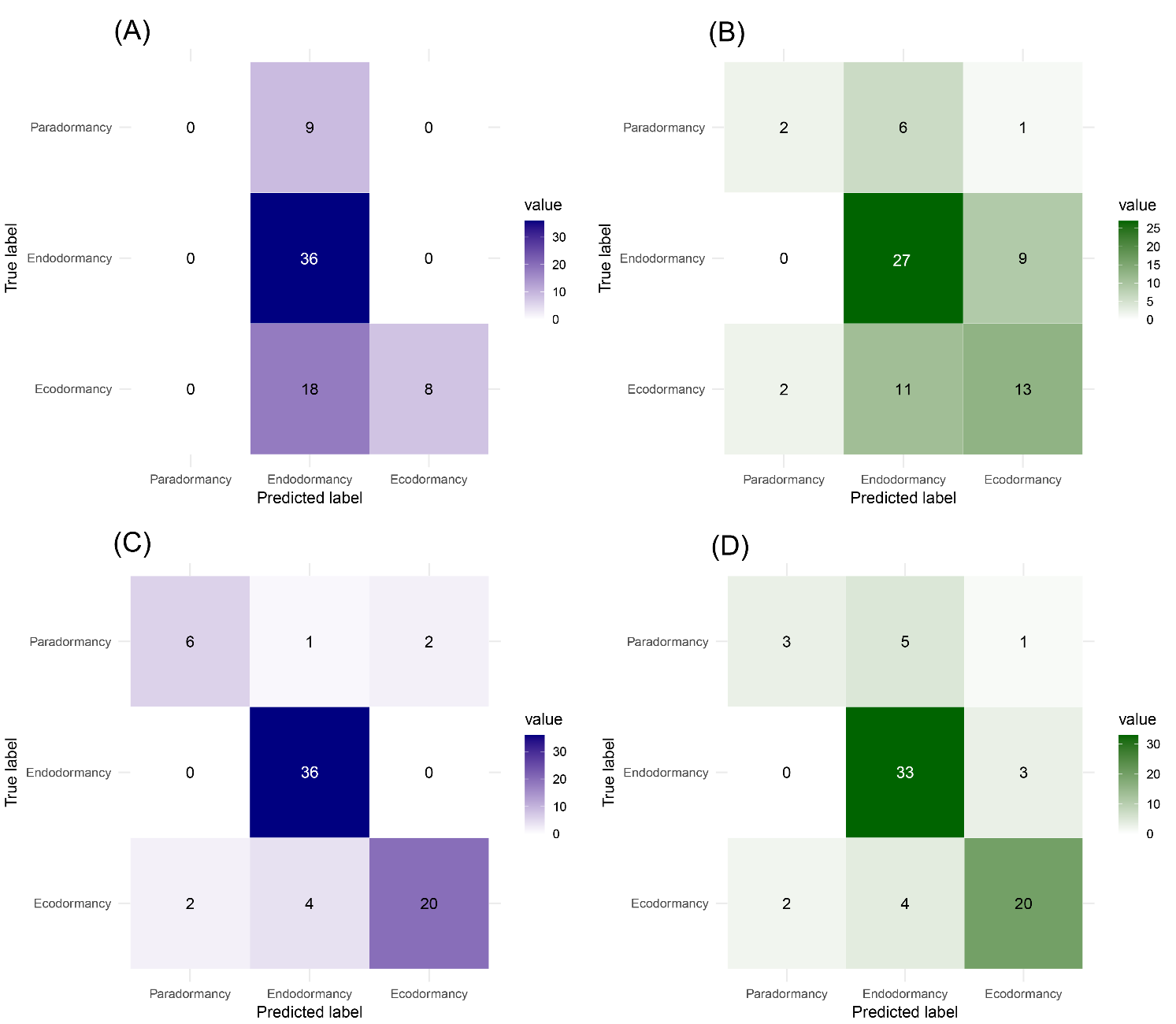


## Figure 2: Confusion matrices of classification models for states dormancy (paradormancy, endodormancy and ecodormancy) through methylation cytosines: (A) Random Forest using all features, (B) XGBoost using all features, (C) Random Forest with selected features, and (D) XGBoost with selected features.


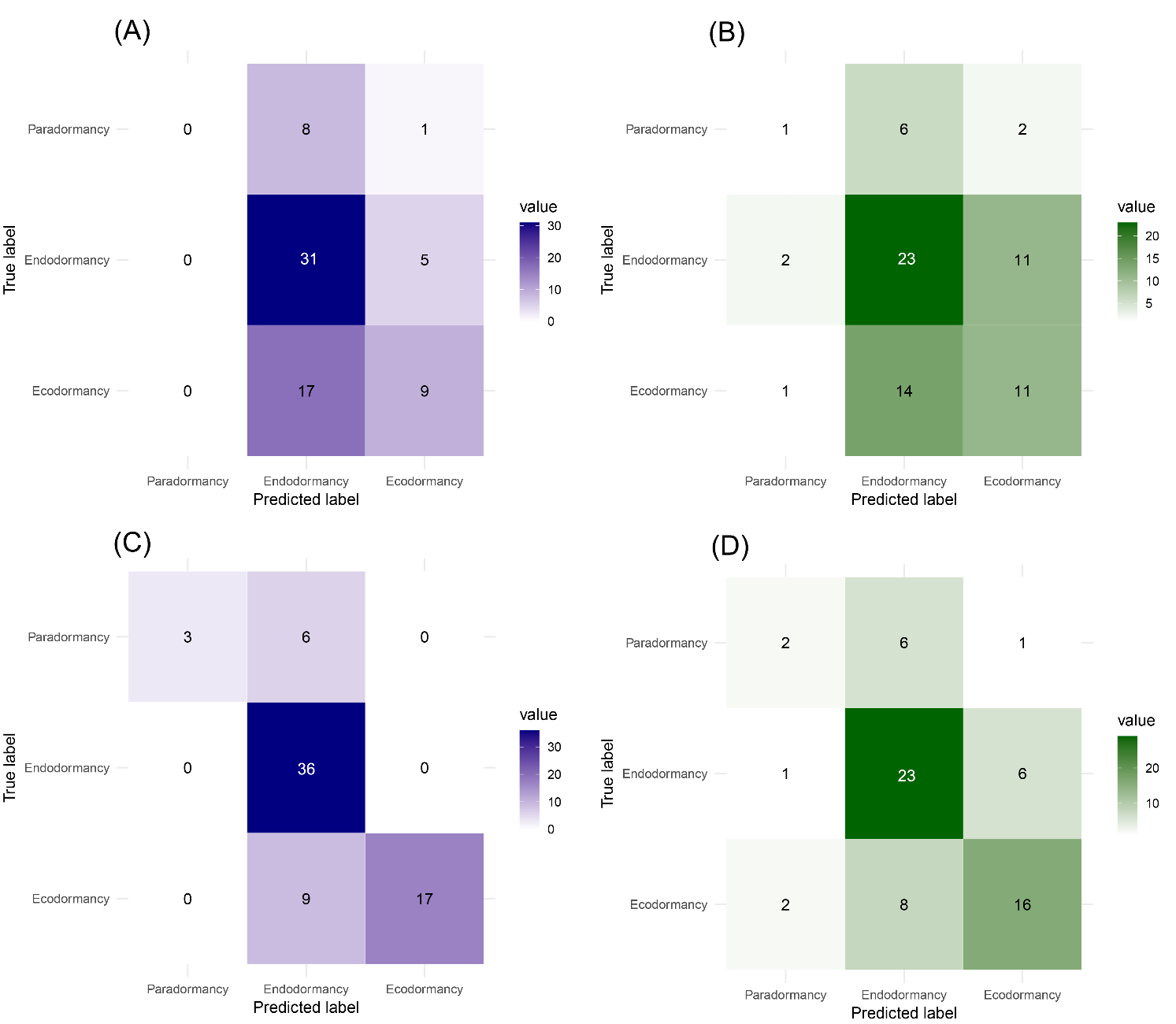


## Figure 3: Confusion matrices of classification models for states dormancy (paradormancy, endodormancy and ecodormancy) through methylation regions: (A) Random Forest using all features, (B) XGBoost using all features, (C) Random Forest with selected features, and (D) XGBoost with selected features.

##
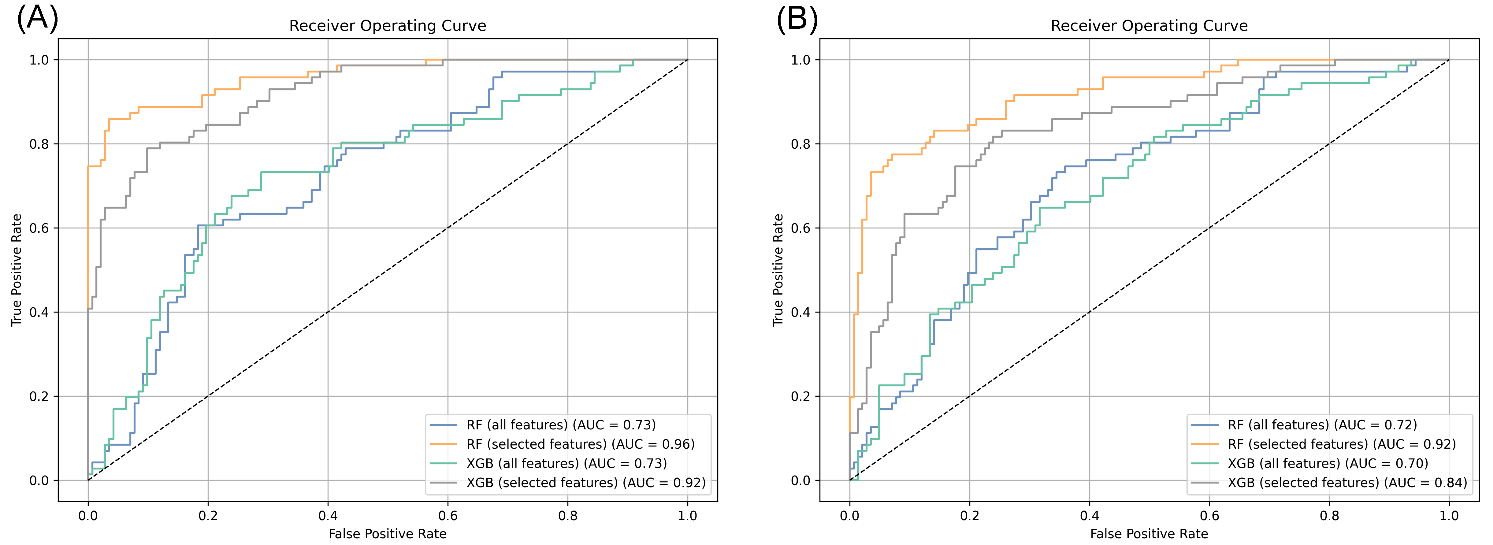


## Figure 4. ROC curves generated to assess the prediction of dormancy stages. (A) Model performance based on methylation cytosine data. (B) Model performance based on methylation region data.

##
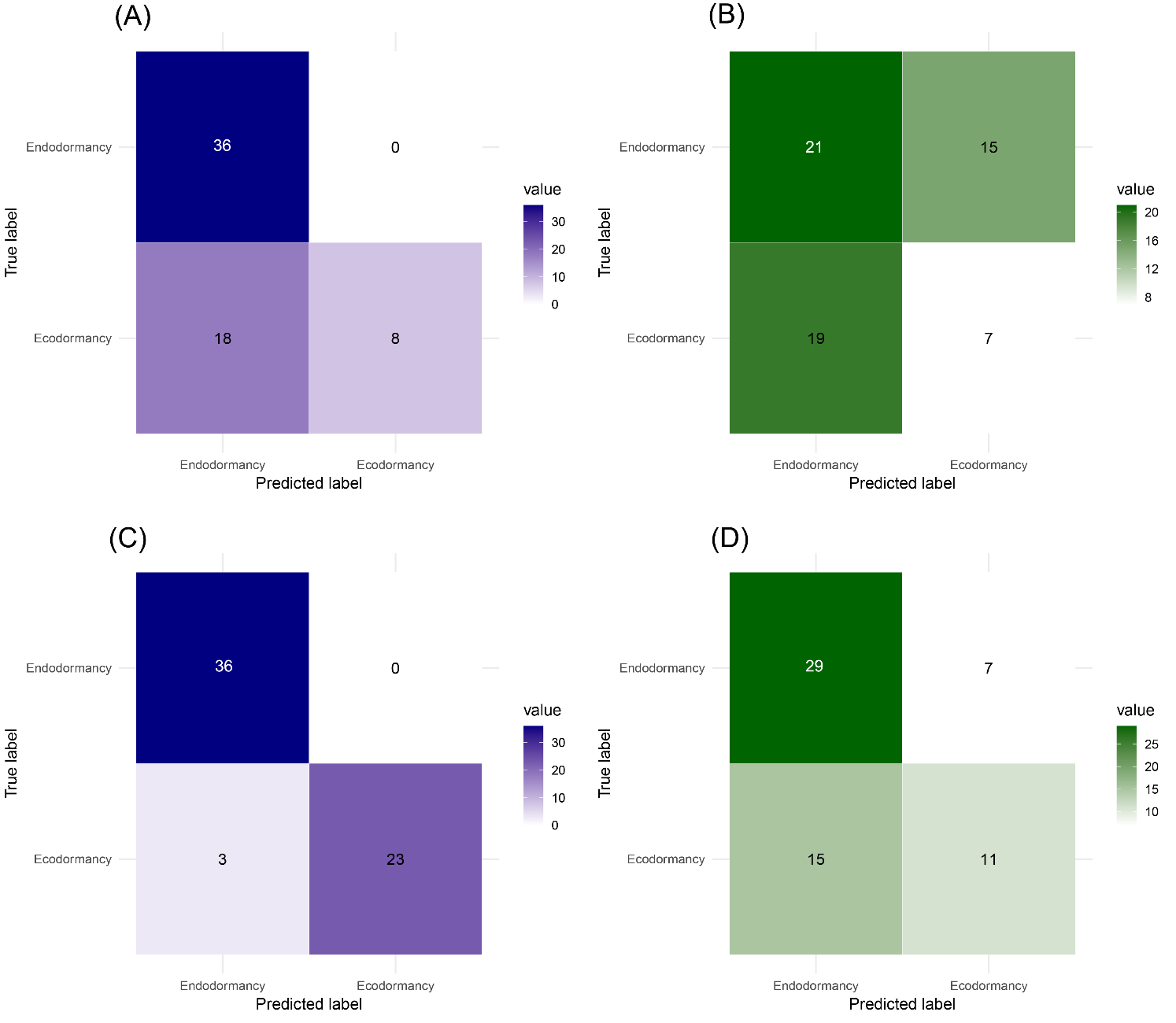


## Figure 5: Confusion matrices of classification models for states dormancy (endodormancy and ecodormancy) through methylation cytosines: (A) Random Forest using all features, (B) XGBoost using all features, (C) Random Forest with selected features, and (D) XGBoost with selected features.


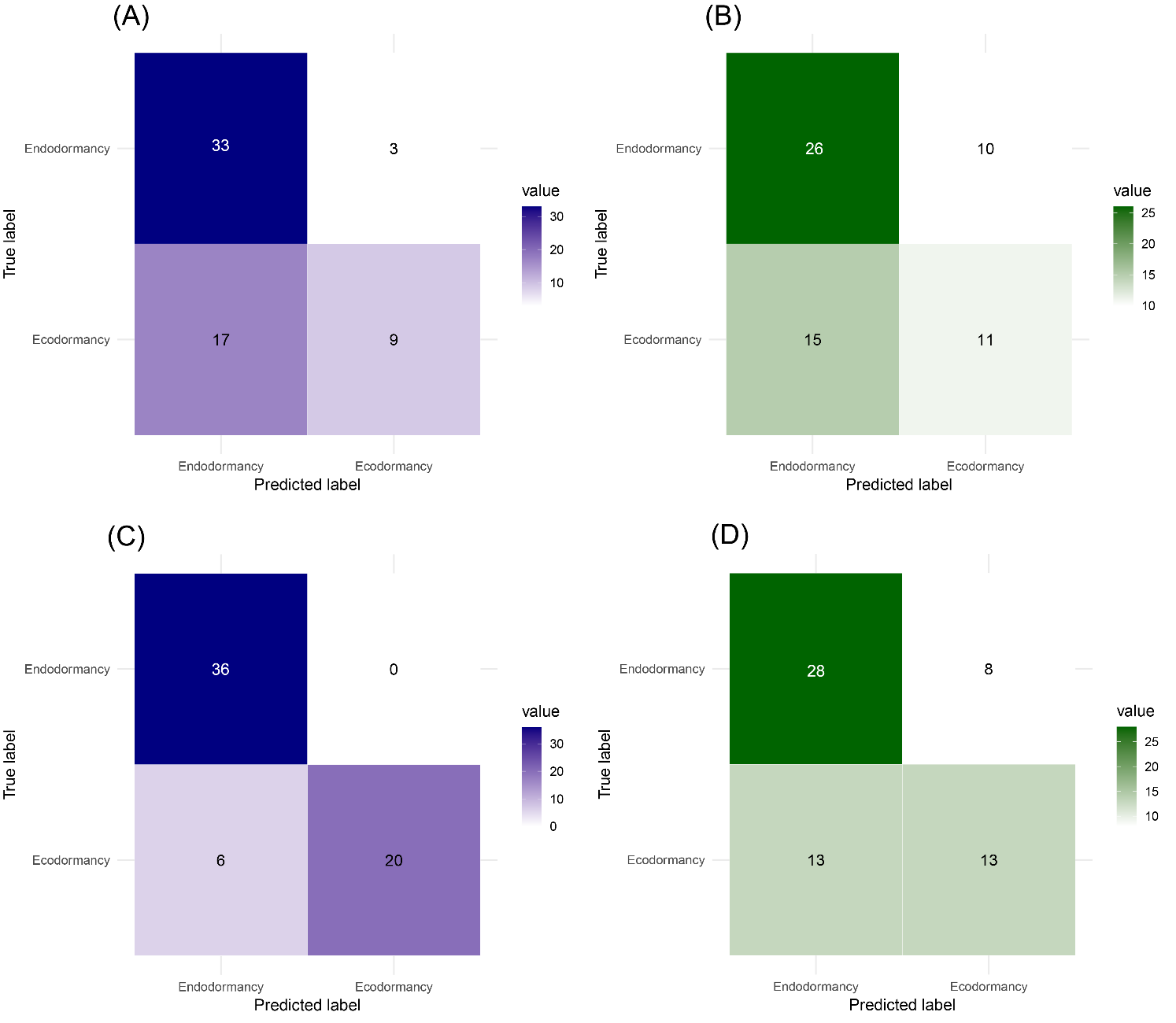


## Figure 6: Confusion matrices of classification models for states dormancy (endodormancy and ecodormancy) through methylation regions: (A) Random Forest using all features, (B) XGBoost using all features, (C) Random Forest with selected features, and (D) XGBoost with selected features.

##
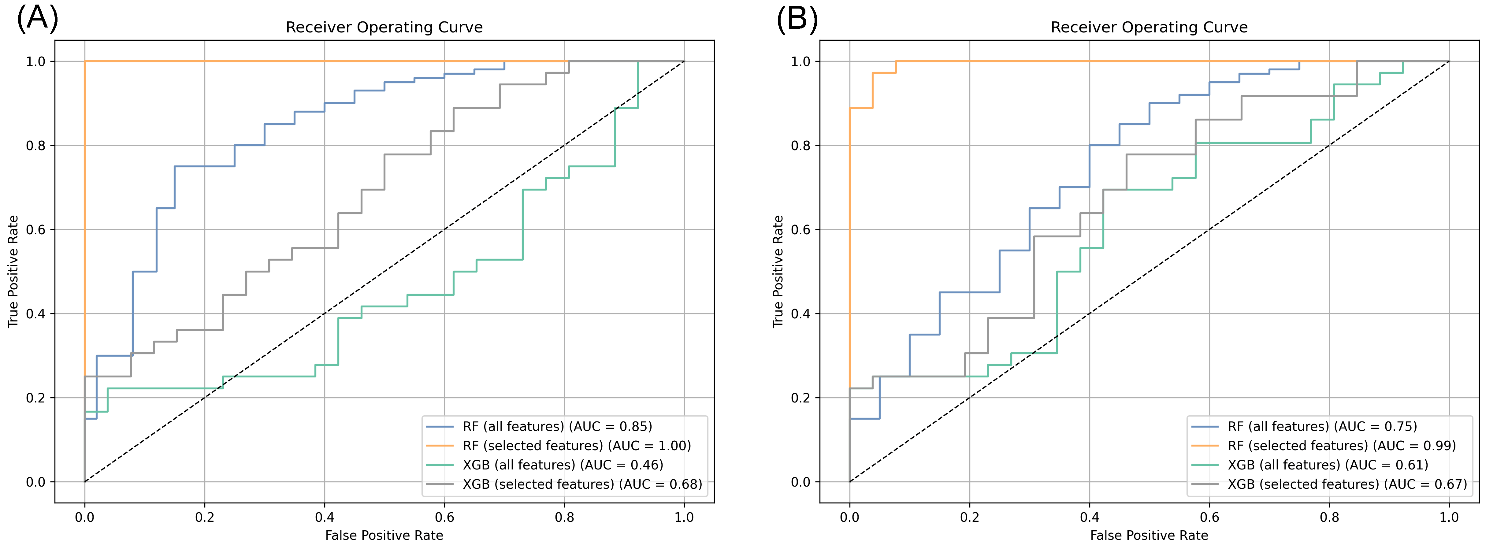


## Figure 7. ROC curves generated to assess the prediction of dormancy stages. (A) Model performance based on methylation cytosine data. (B) Model performance based on methylation region data.

##
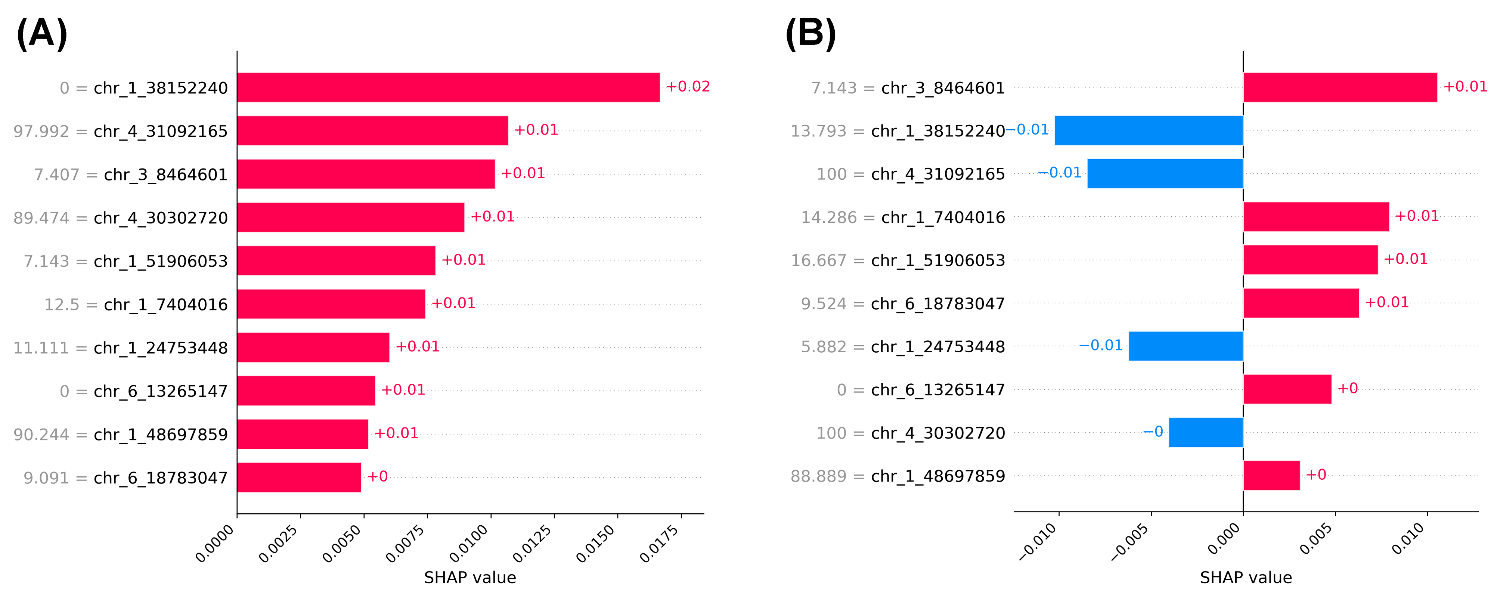
Figure 8: SHAP force plots of feature-level impact for selected samples. (A) Most impactful features for a correctly classified ecodormancy sample. (B) Feature contributions for a misclassified sample. Highlighting features that increased (red) or decreased (blue) the predicted probability.


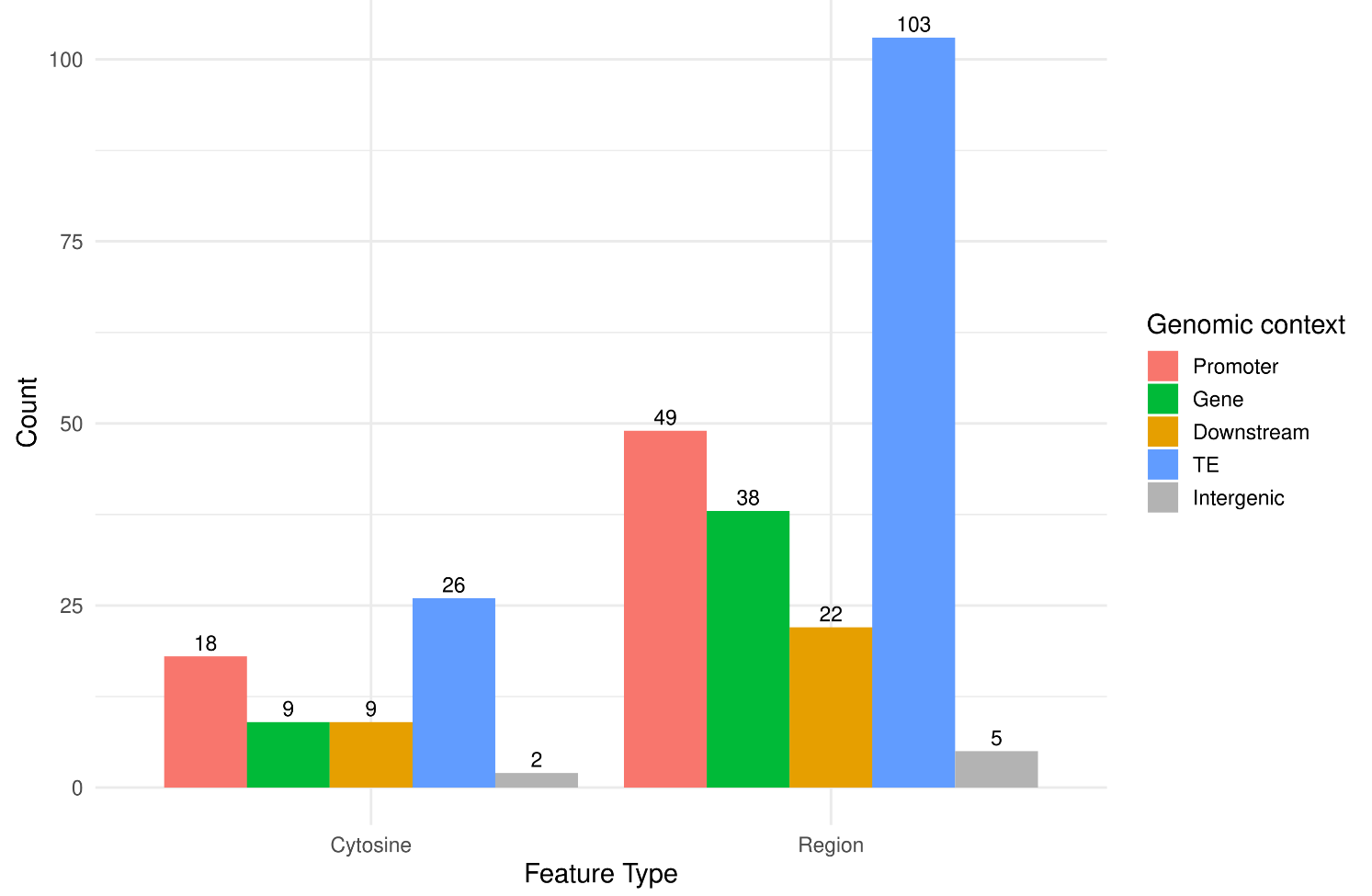


## Figure 9: Genomic annotation of relevant cytosines and regions in the 3-stages model. Cytosine-level and region-level features were detected in the model in five genomic contexts: promoter (2 kb upstream of TSS), gene bodies (exonic and intronic regions), downstream (2 kb downstream 3'), transposable element (TE), and intergenic space (regions not overlapping annotated gene-proximal features).


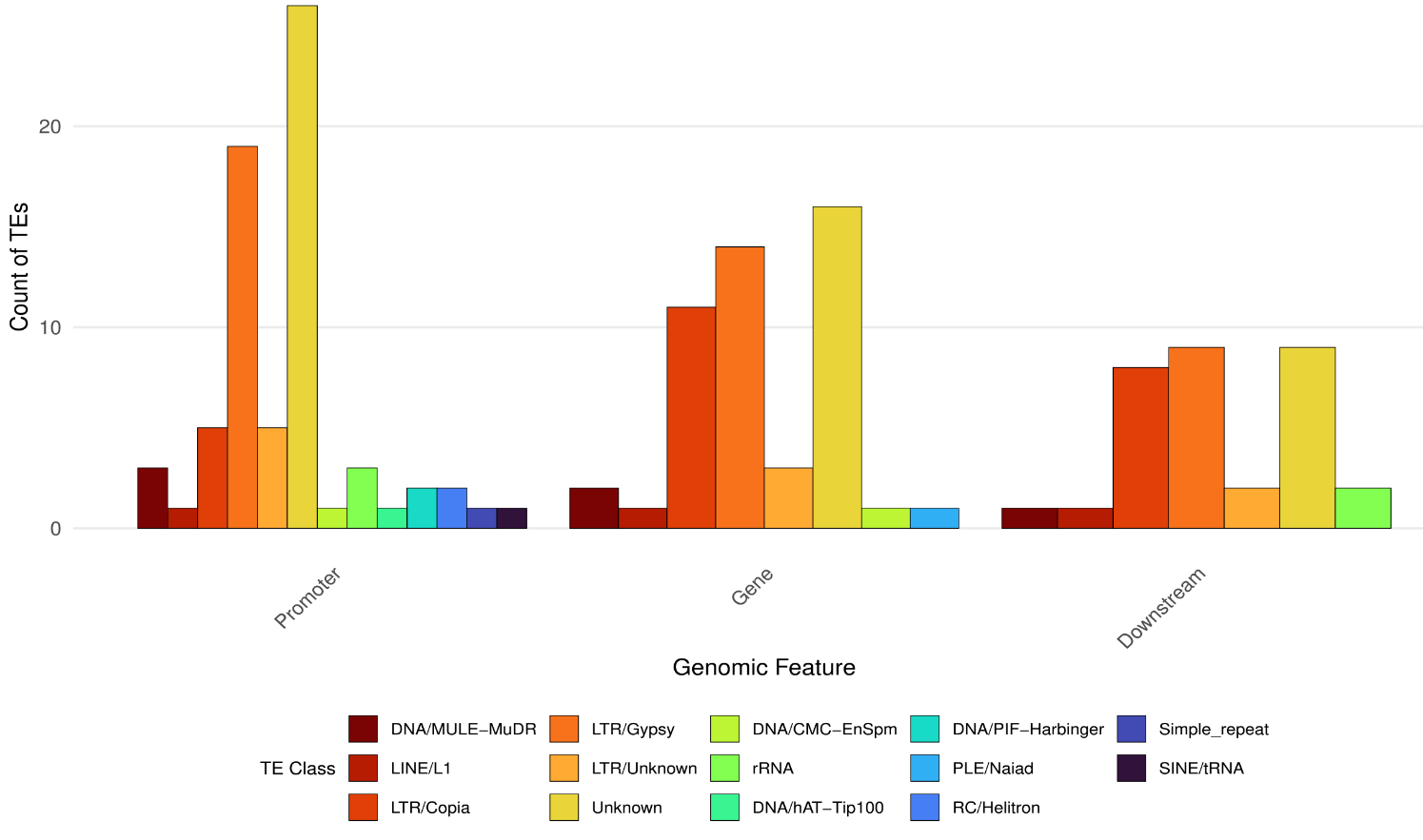


## Figure 10: Class-wise distribution of features from model 3 stages overlapping TEs and their genomic context (Promoter, Gene, and Downstream). TE class abbreviations are as follows: DNA/MULE-MuDR (Mutator-like elements, a family of DNA transposons), LINE/L1 (long interspersed nuclear elements of the LINE-1 family), LTR/Copia (LTR retrotransposons of the Copia superfamily), LTR/Gypsy (long terminal repeat retrotransposons of the Gypsy superfamily), LTR/Unknown (putative LTR retrotransposons not assigned to a known superfamily), Unknown (unclassified or unknown transposable elements), DNA/CMC-EnSpm (CACTA, Mirage, and Chapaev elements of the En/Spm superfamily of DNA transposons), rRNA (ribosomal RNA repeat regions), DNA/hAT-Tip100 (Tip100 subfamily of the hAT transposons), DNA/PIF-Harbinger (P instability factor transposons of the Harbinger superfamily), PLE/Naiad (Penelope-like elements of the Naiad subfamily), RC/Helitron (rolling-circle DNA transposons of the Helitron family), Simple_repeat (tandemly repeated microsatellite or simple sequence repeats), and SINE/tRNA (short interspersed nuclear elements derived from tRNA).


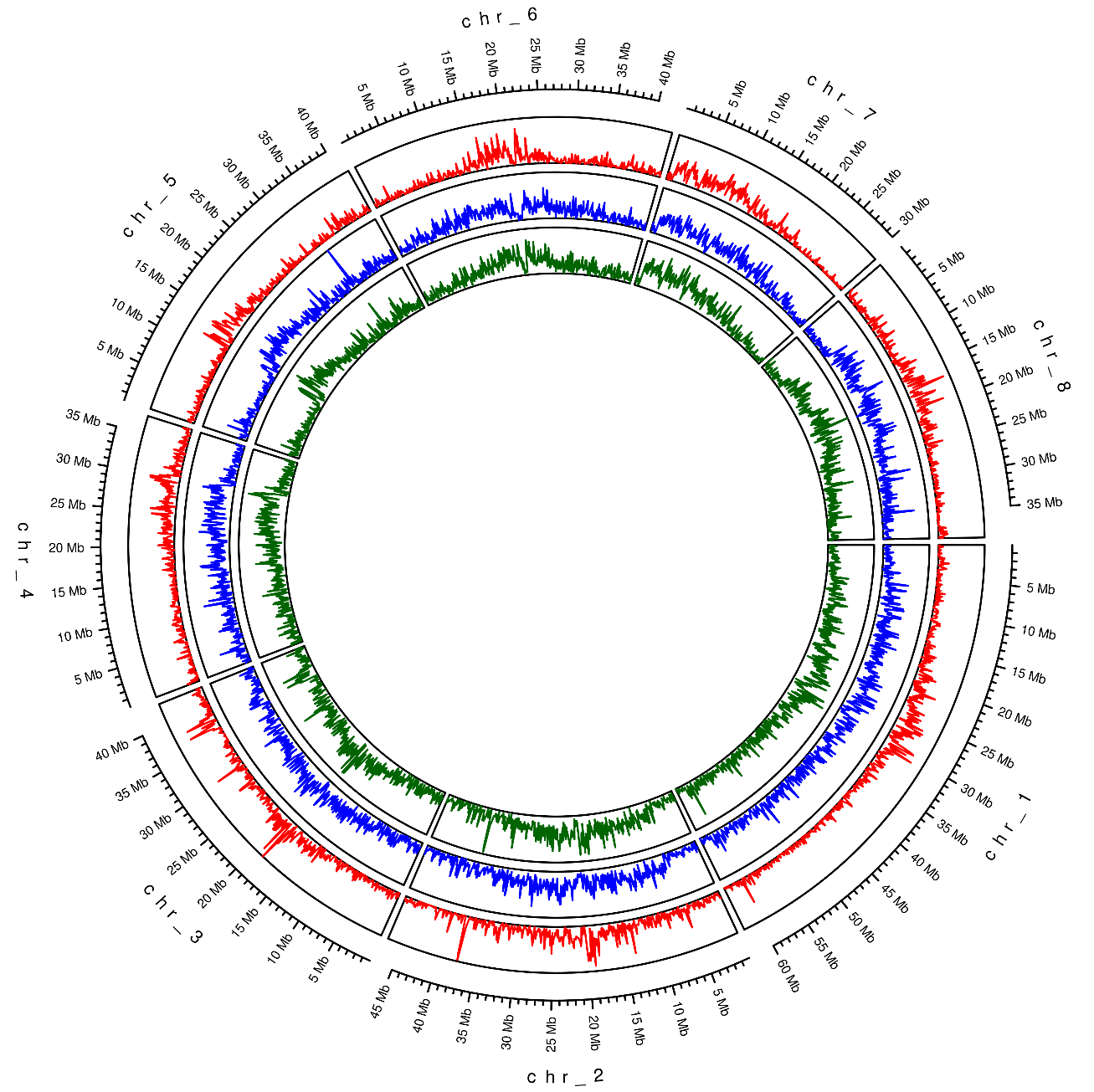


## Figure 11: Description of the bisulfite sequencing (BS-Seq) data of all paired-end libraries individually. Quality statistics of raw and after trimming datasets.

# Supplementary codes

## Code 1: Pseudocode for feature importance selection and classification performance evaluation

cv = StratifiedKFold(n_splits=10, shuffle=True, random_state=42)

for train_idx, test_idx in cv.split(data,stage):

#creation of training and testing datasets

X_train, X_test = data.iloc[train_idx], data.iloc[test_idx]

y_train, y_test = stage.iloc[train_idx], stage.iloc[test_idx]

#initialize the models

model_rf = RandomForestClassifier(n_estimators=10000)

model_xgb = xgb.XGBClassifier(n_estimators=10000, learning_rate=0.05, reg_alpha=0.1, reg_lambda=0.1, eval_metric='mlogloss')

#training models

model_rf.fit(X_train, y_train)

model_xgb.fit(X_train, y_train)

#performance metrics

y_pred_rf = model_rf.predict(X_test)

y_pred_xgb = model_xgb.predict(X_test)

accuracy_rf_list.append(accuracy_score(y_test, y_pred_rf))

accuracy_xgb_list.append(accuracy_score(y_test, y_pred_xgb))

f1_rf_list.append(f1_score(y_test, y_pred_rf, average='weighted'))

f1_xgb_list.append(f1_score(y_test, y_pred_xgb, average='weighted'))

precision_rf_list.append(precision_score(y_test, y_pred_rf, average='weighted'))

precision_xgb_list.append(precision_score(y_test, y_pred_xgb, average='weighted'))

recall_rf_list.append(recall_score(y_test, y_pred_rf, average='weighted'))

recall_xgb_list.append(recall_score(y_test, y_pred_xgb, average='weighted'))

print(f"Random Forest - Accuracy: {np.mean(accuracy_rf_list):.4f}, F1-score: {np.mean(f1_rf_list):.4f}, "f"Precision: {np.mean(precision_rf_list):.4f}, Recall: {np.mean(recall_rf_list):.4f}")

print(f"XGBoost - Accuracy: {np.mean(accuracy_xgb_list):.4f}, F1-score: {np.mean(f1_xgb_list):.4f}, "f"Precision: {np.mean(precision_xgb_list):.4f}, Recall: {np.mean(recall_xgb_list):.4f}")

#calculate RF and XGboost importances

mdi_importances_rf = model_rf.feature_importances_

mdi_importances_rf_list.append(mdi_importances_rf)

gain_importance_xgb = model_xgb.get_booster().get_score(importance_type='gain')

feature_importance_dict = {col: 0 for col in X_train.columns}

feature_importance_dict.update(gain_importance_xgb)

importance_array = np.array(list(feature_importance_dict.values()))

xgb_importances_list.append(importance_array)

#calculate RF and XGboost importances with SHAP

explainer_rf = shap.Explainer(model_rf, X_train)

shap_values_rf = explainer_rf(X_train, check_additivity=False)

shap_importances_rf = np.abs(shap_values_rf.values).mean(axis=0)

average_importance_rf = shap_importances_rf.mean(axis=1)

shap_importances_rf_list.append(average_importance_rf)

explainer_xgb = shap.Explainer(model_xgb, X_train)

shap_values_xgb = explainer_xgb(X_train)

shap_importances_xgb = np.abs(shap_values_xgb.values).mean(axis=0)

average_shap_importances_xgb = shap_importances_xgb

shap_importances_xgb_list.append(shap_importances_xgb)

y_true_all.extend(y_test)

y_pred_rf_all.extend(y_pred_rf)

y_pred_xgb_all.extend(y_pred_xgb)

#average feature importance for RF

shap_importances_rf_mean = np.mean(shap_importances_rf_list, axis=0)

mdi_importances_rf_mean = np.mean(mdi_importances_rf_list, axis=0)

shap_importances_xgb_mean = np.mean(shap_importances_xgb_list, axis=0)

shap_importances_xgb_mean = shap_importances_xgb_mean

xgb_importances_mean = np.mean(xgb_importances_list, axis=0)

#create dataframe with results

importance_df = pd.DataFrame({

'Feature': X.columns,

'RF SHAP_Importance': shap_importances_rf_mean,

'RF MDI_Importance': mdi_importances_rf_mean,

'XGB SHAP_Importance': shap_importances_xgb_mean,

'XGB Importance': xgb_importances_mean

})

scaler = MinMaxScaler()

importance_df_normalized = pd.DataFrame(scaler.fit_transform(importance_df.iloc[:,1:5]))

importance_final = pd.DataFrame({

'Feature': X.columns,

'RF SHAP_Importance': importance_df_normalized[0],

'RF MDI_Importance': importance_df_normalized[1],

'XGB SHAP_Importance': importance_df_normalized[2],

'XGB Importance': importance_df_normalized[3]

})

importance_final.to_csv("out.txt", sep='\t', index=False)
